# *SERPINB2* is a novel TGFβ-responsive lineage fate determinant of human bone marrow stromal cells

**DOI:** 10.1038/s41598-017-10983-x

**Published:** 2017-09-07

**Authors:** Mona Elsafadi, Muthurangan Manikandan, Muhammad Atteya, Raed Abu Dawud, Sami Almalki, Zahid Ali Kaimkhani, Abdullah Aldahmash, Nehad M. Alajez, Musaad Alfayez, Moustapha Kassem, Amer Mahmood

**Affiliations:** 1 Stem Cell Unit, Department of Anatomy, College of Medicine, King Saud University, Riyadh, Saudi Arabia; 2 KMEB, Department of Endocrinology, University Hospital of Odense and University of Southern Denmark, Odense, Denmark; 3 Department of Histology, Faculty of Medicine, Cairo University, Cairo, Egypt; 4 Berlin-Brandenburg Center for Regenerative Therapies (BCRT), Charité-Universitätsmedizin Berlin, Berlin, Germany; 5 Department of Comparative Medicine, King Faisal Specialist Hospital and Research Centre, Riyadh, Saudi Arabia; 6 College of Agriculture, King Saud University, Riyadh, Saudi Arabia; 7 Prince Naif Health Research Center, King Saud University, Riyadh, 11461 Saudi Arabia

## Abstract

TGF-β1, a multifunctional regulator of cell growth and differentiation, is the most abundant bone matrix growth factor. During differentiation of human bone stromal cells (hBMSCs), which constitute bone marrow osteoblast (OS) and adipocyte (AD) progenitor cells, continuous TGF-β1 (10 ng/ml) treatment enhanced OS differentiation as evidenced by increased mineralised matrix production. Conversely, pulsed TGF-β1 administration during the commitment phase increased mature lipid-filled adipocyte numbers. Global gene expression analysis using DNA microarrays in hBMSCs treated with TGF-β1 identified 1587 up- and 1716 down-regulated genes in OS-induced, TGF-β1-treated compared to OS-induced hBMSCs (2.0 fold change (FC), p < 0.05). Gene ontology (GO) analysis revealed enrichment in ‘osteoblast differentiation’ and ‘skeletal system development-associated’ genes and up-regulation of several genes involved in ‘osteoblastic-differentiation related signalling pathways’. In AD-induced, TGF-β1-treated compared to AD-induced hBMSCs, we identified 323 up- and 369 down-regulated genes (2.0 FC, p < 0.05) associated with ‘fat cell differentiation’, ‘fatty acid derivative biosynthesis process’, ‘fatty acid derivative metabolic process’, and ‘inositol lipid-mediated’. Serpin peptidase inhibitor, clade B (ovalbumin), member 2 (*SERPINB2*) was down-regulated 3-fold in TGF-β1-treated hBMSCs. siRNA-mediated SERPINB2 inhibition enhanced OS and AD differentiation. Thus, TGF-β signalling is important for hBMSC OS and AD differentiation and *SERPINB2* is a TGF-β-responsive gene that plays a negative regulatory role in hBMSC differentiation.

## Introduction

Skeletal stem cells (also known as bone marrow-derived multipotent stromal cells or bone marrow mesenchymal stem cells (BMSC)) comprise multipotent stem cells that can differentiate into adipocytes (ADs or osteoblasts (OS) in response to micro-environmental signals including growth factors, cytokines, and epigenetic regulators^1^. An imbalance between OS and AD lineage commitment and differentiation has been implicated as a cause for age-related impaired bone formation; thus, a number of therapeutic interventions have been proposed for enhancing bone mass through the targeting of BMSC^2, 3^.

TGF-β1 constitutes one of the most abundant growth factor in the bone matrix (200 μg/kg)^4^ and is secreted by several cells associated with the skeleton; e.g. OS, endothelial cells, smooth muscle cells, and stromal cells, as well as by cells of the immune system^5^. TGF-β1 is produced in large precursor complexes that are composed of mature TGF-β1 and latency-associated protein (LAP). TGF-β1 is secreted and deposited in bone matrix as an inactive, latent complex owing to its non-covalent linkage to LAP, which masks the receptor-domains of the active TGF-β1. Osteoclast-mediated bone resorption activates TGF-β1 by cleavage of LAP and releases it from the bone matrix, creating a gradient of active TGF-β1 that signals to recruit osteoprogenitor cells to the bone remodelling sites and thus support bone formation^6^. TGF-β1 has been shown to regulate the proliferation and differentiation of osteoblastic cells^7^ and inhibition of TGF-β receptor signalling in OS has been reported to decrease bone remodelling and increase trabecular bone mass^6^.

In the current study, we examined the role of TGFβ-1 in OS and AD lineage commitment and the differentiation of human BMSC (hBMSC) and the dependency of these effects on the timing of induction as determined using a single pulse dose during the commitment phase of hBMSC versus continuous treatment during the whole differentiation period. In addition, we examined the molecular mechanisms of TGFβ-1-mediated differentiation of hBMSCs employing DNA microarrays. We identified one of the significantly (3-fold) down-regulated genes during TGFβ1 stimulation, serpin peptidase inhibitor, clade B (ovalbumin), member 2 (SERPINB2), as a novel TGF-β-responsive gene that plays a role in hBMSC differentiation. We demonstrated that inhibition of SERPINB2 in hBMSC led to enhanced OS and AD differentiation suggesting a negative regulatory role in OS and AD differentiation, downstream of TGF-β signalling.

## Results

### Continuous treatment with TGF-β1 enhances OS differentiation

**Figure 1:**
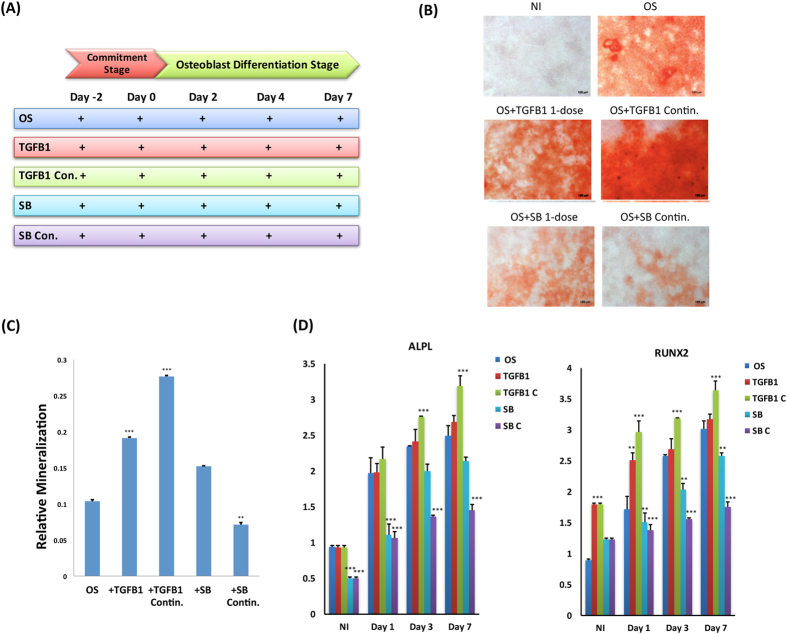
TGF-β1 promotes osteogenic differentiation. Human bone marrow stromal (skeletal) stem cells (hBMSC) were differentiated into osteoblasts (OS) using osteogenic induction mixture for 7 days. (**A**) Time line scheme of experimental setup illustrating TGF-β1 or SB-431542 (SB) treatment that was performed as either single pulse dose (TGFB1 1-dose or SB 1-dose) or continuous treatment (TGFB1 Contin. Or SB Contin.) at commitment and differentiation stages of *in vitro* OS differentiation **(B)** Mineralised calcium deposition was determined by Alizarin Red S staining, which is shown as microscopic images (20× magnification). **(C)** Alizarin Red Quantification under different experimental conditions: osteo-induced (OS), single dose of 10 ng/ml TGF-β1 (+TGFB1); continuous exposure to TGF-β1, TGFB1 con, SB 1-dose, and SB Con. Data are presented as the means ± SD of three independent experiments; *n* = 6; **(D)** qRT-PCR of *ALPL* (left panel) or *RUNX2* (right panel) mRNA expression preformed on cells exposed to the indicated treatment on days 1, 3, and 7. Expression of each target gene was normalised to *GAPDH*. Data are presented as the means ± SD from three independent experiments, *n* = 6; *p < 0.05; **p < 0.01, ***p < 0.005.

We compared the effect on hBMSC differentiation to OS when TGFβ1 (10 ng/ml) treatment was conducted as a single pulse dose during the commitment phase of differentiation (day −2 to day 0) versus continuous treatment during the whole course of differentiation (day −2 to day 7) (Fig. 1A). As judged by qualitative and quantitative alizarin red staining for mineralised matrix formation, continuous treatment with TGF-β1 induced a higher degree of OS differentiation (Fig. 1B,C, p < 0.01). These effects represented direct effects of TGF-β1, as they were reduced following exposure to the TGFβ receptor kinase inhibitor SB-431542 (10 M). Quantitative reverse transcription-polymerase chain reaction (RT-PCR) was further performed to assess gene expression of osteoblastic markers upon continuous application of TGFβ-1. Gene expression of alkaline phosphatase, liver/bone/kidney (ALPL) exhibited significant up-regulation at day 3, whereas runt-related transcription factor 2 (RUNX2) showed gradual up-regulation starting from day 1 up to day 3 (Fig. 1D).

### Single pulse treatment with TGFβ-1 enhances AD differentiation

**Figure 2:**
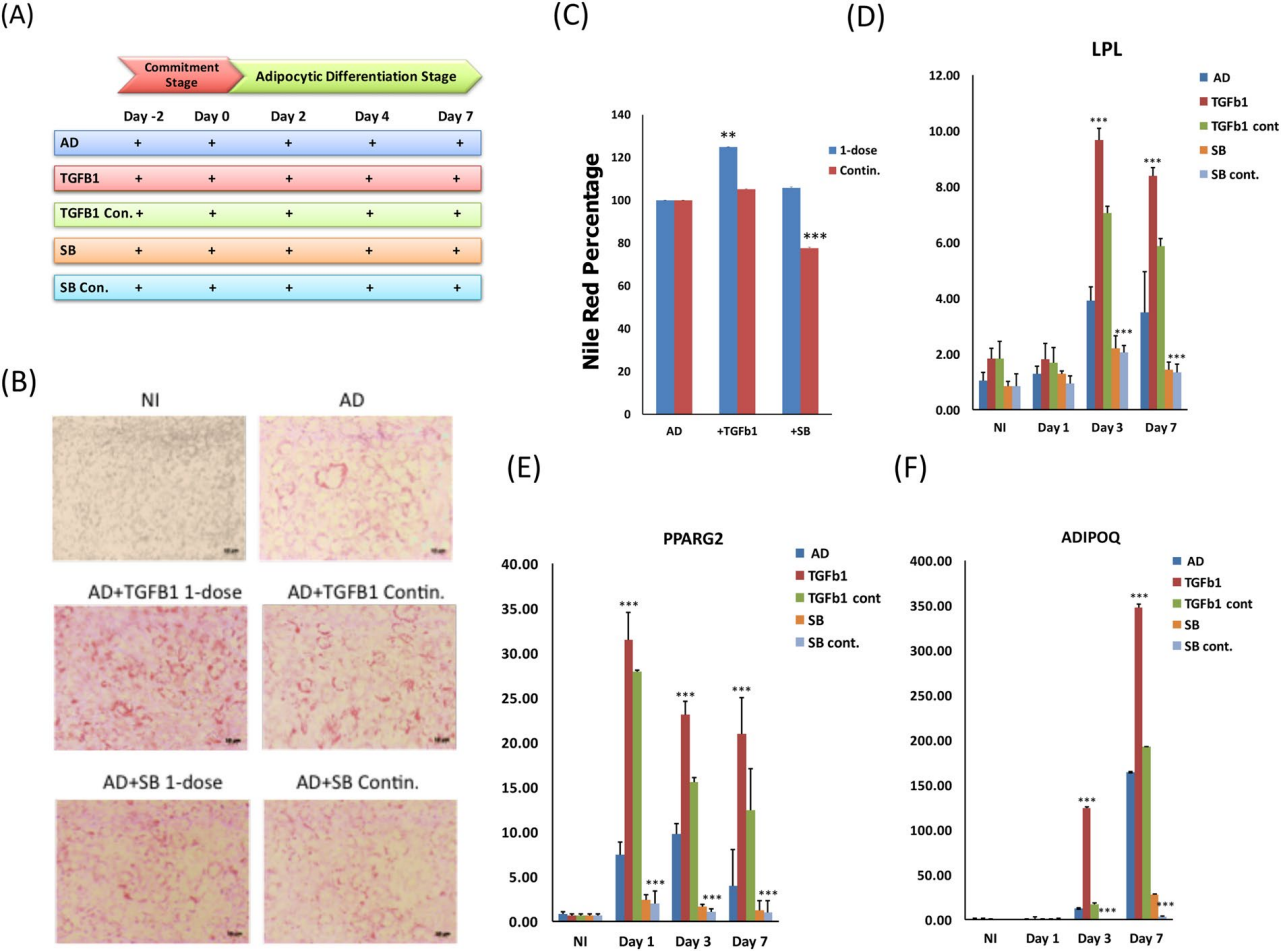
TGF-β1 promotes adipogenic differentiation. **(A)** Time line schematic model illustrating the dose of TGF-β1 or SB-431542 (SB) treatment for either single pulse or continuous treatment at commitment and differentiation stages during adipogenic induction of human bone marrow stromal (skeletal) stem cells (hBMSC) **(B)** hBMSCs were induced into adipocyte using adipocyte induction medium in the presence of single dose (AD + TGFB1 1-dose) or continuous (AD + TGFB1 Contin.) exposure to 10 ng/ml TGF-β1 or a single (AD + SB 1-dose) or continuous (AD + SB Contin.) treatment with 10 µM SB-431542. Cells were stained on day 7 using oil red O staining for adipocytes containing lipid droplets, and shown as microscopic images (20×, magnification), non-induced (NI), adipocytic induced (AD). **(C)** Nile red quantification under the indicated treatment conditions was performed on day 7 post adipocyte induction. qRT-PCR quantification for *LPL* **(D)**, *PPARG2* **(E)**, and *ADIPOQ* **(F)** mRNA under the indicated experimental conditions: non-induced (CNT), adipo-induced (AD), single dose of TGF-β1 (+TGFB1), continuous exposure to TGF-β1 (+TGFB1 Contin.), single dose of SB-431542 (+SB), and continuous dose of SB-431542 (+SB Contin.). Expression of each target gene was normalised to *GAPDH*. Data are presented as the means ± SD from three independent experiments, *n* = 6; *p < 0.05; **p < 0.01, ***p < 0.005.

We next compared the effect on hBMSC differentiation to AD when TGFβ1 (10 ng/ml) treatment was conducted as a single pulse dose during the commitment phase of differentiation (day −2 to day 0) versus continuous treatment during the whole course of differentiation (day −2 to day 7) (Fig. 2A). As shown in Fig. 2A and B, qualitative and quantitative Nile red staining of mature adipocytes following single pulse treatment with TGFβ-1 led to a greater degree of AD differentiation enhancement compared with continuous treatment (Fig. 2B and C). SB-431542 treatment significantly reduced AD induction during continuous treatment and to a lesser degree in the single pulse dose. Gene expression analysis of the adipogenic markers lipoprotein lipase (*LPL*), peroxisome proliferator-activated receptor gamma 2 (*PPARG2*), and adiponectin or (*ADIPOQ)* exhibited similar effects (Fig. 2D–F).

### Molecular phenotype of TGF-β1-induces osteoblastic cells

**Figure 3:**
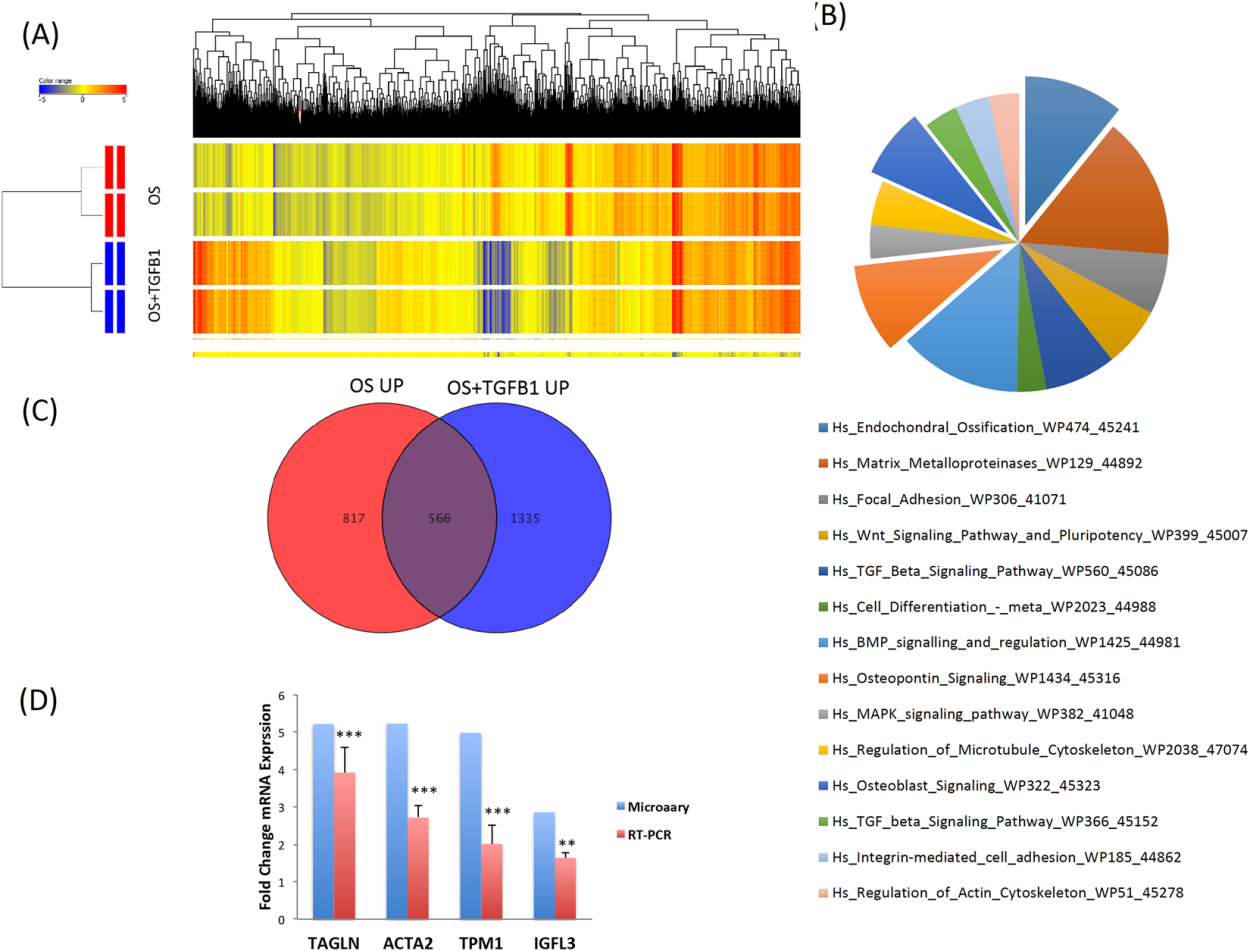
Gene expression profiling on hBMSC induced into osteoblasts in the presence of TGF-β1. **(A)** Hierarchical clustering of human bone marrow stromal (skeletal) stem cells (hBMSC) induced into osteoblasts (OS) (day 3) in the presence or absence of TGF-βl. Each row represents one replica sample and each column represents a transcript. Expression level of each gene in a single sample is depicted according to the colour scale. **(B)** Pie chart illustrating the distribution of the top 13 pathway designations for the up-regulated genes in TGF-β1 treated cells during OS differentiation **(C)** Venn diagram depicting the overlap between the up-regulated genes during OS differentiation of hBMSCs and the upregulated genes in hBMSC induced to OS in presence of TGF-β1. **(D)** qRT-PCR validation of selected genes (*TAGLN1*, *ACTA2*, *TPM1*, and *IGFL3*).

Global gene expression profiling was conducted on hBMSC treated with or without TGF-β1 (10 ng/ml) in the presence of OS induction medium. Hierarchical clustering based on differentially expressed genes revealed clear separation of control cells treated with OS induction medium alone (OS-differentiated) from those treated with TGF-βl (OS-differentiated-TGF-β1) (Fig. 3A). We identified 1587 up-regulated and 1716 down-regulated genes in OS-differentiated-TGF-β1 compared to OS-differentiated control (2.0 fold change (FC), p < 0.05; Supplementary Tables S1, S2). Pathway analysis of the up-regulated genes revealed significant enrichment in several pathways related to ‘endochondral ossification’, ‘matrix metalloproteinases’, ‘TGF-β signalling’, ‘WNT signalling’, ‘MAPK signalling’, ‘focal adhesion’, and’ regulation of actin cytoskeleton pathways’ (Fig. 3B, Supplementary Figs S1–S7). Table 1 lists the matched entities from the microarray data and the corresponding signalling pathway. Comparing the up-regulated genes in OS-differentiated cells with those in OS-differentiated-TGF-β1 cells revealed 566 common genes that were predicted to be regulated by TGF-β signalling and concurrently to be involved in OS-differentiation (Fig. 3C, Supplementary Table S3). Notably, we found that the selectively up-regulated genes in OS-differentiated-TGF-β1 cells were enriched in categories of bone formation, such as ‘skeletal system development’ (58 genes), ‘ossification’ (42 genes), and ‘osteoblast differentiation’ (23 genes) (Table 2). qRT-PCR results for selective up-regulated osteoblastic-related genes showed good concordance with microarray results (Fig. 3D).

**Table 1:** Up-regulated genes involved in osteogenic-related pathways in TGF-β treated cells after osteogenic induction.

Endochondral Ossification	Matrix Metallo-proteinases	TGFB Signalling	WNT Signalling	MAPK Signalling	Focal Adhesion	Regulation of Actin Cytoskeleton
*IGF1*	*MMP1*	*INHBA*	*WNT7B*	*IL1A*	*COL11A1*	*ITGA1*
*IGF1R*	*MMP2*	*NOG*	*WNT11*	*TGFB2*	*COL3A1*	*GSN*
*IGF2*	*MMP11*	*THBS1*	*FZD2*	*ACVR1C*	*COL4A1*	*NRAS*
*BMP6*	*MMP14*	*LEFTY1*	*FZD7*	*CASP9*	*COL4A2*	*IGF1*
*CDKN1C*	*MMP24*	*SMAD7*	*DVL1*	*AKT3*	*COL4A4*	*PDGFA*
*TGFB2*	*MMP23B*	*LIF*	*CTNNB1*	*HSPB2*	*COL5A1*	*C11orf13*
*COL10A1*	*TIMP1*	*CTNNB1*	*TCF7*	*MAPK8*	*COL5A2*	*BDKRB1*
*C4ST1*	*TIMP2*	*RUNX3*	*TCF7L1*	*HSPA5*	*COL1A1*	*LIMK1*
*RUNX3*	*TIMP3*		*NKD1*	*MAPK8IP3*	*THBS1*	*PAK3*
*TIMP3*			*PPP2R1B*	*MAP3K14*	*THBS2*	
*PLAT*			*PLAU*	*NRAS*	*TNC*	
*PLAU*			*PPARD*		*ARHGAP5*	
*ADAMTS4*					*AKT3*	
					*PAK3*	
					*MAPK8*	
					*IGF1R*	
					*ITGB1*	
					*ITGB3*	
					*ITGA11*	
					*IGF1*	
					*PDGFA*	
					*PDGFC*	
					*PGF*

**Table 2:** Up-regulated biological processes and related genes in TGF-β1 treated cells during osteogenesis using GO analysis.

Skeletal System Development		Ossification		Osteoblast Differentiation	
Gene Symbol	Gene Name	Gene Symbol	Gene Name	Gene Symbol	Gene Name
*CYTL1*	cytokine-like 1	*GLI1*	GLI family zinc finger 1	*GLI1*	GLI family zinc finger 1
*HOXB2*	homeobox B2	*SEMA7A*	semaphorin 7 A, GPI membrane anchor (John Milton Hagen blood group)	*SEMA7A*	semaphorin 7 A, GPI membrane anchor (John Milton Hagen blood group)
*PLEKHA1*	pleckstrin homology domain containing, family A (phosphoinositide binding specific) member 1	*FBXL15*	F-box and leucine-rich repeat protein 15	*SNAI1*	snail family zinc finger 1
*CSRNP1*	cysteine-serine-rich nuclear protein 1	*SNAI1*	snail family zinc finger 1	*VCAN*	versican
*SNAI1*	snail family zinc finger 1	*IGF1*	insulin-like growth factor 1 (somatomedin C)	*IGF2*	insulin-like growth factor 2 (somatomedin A)
*IGF1*	insulin-like growth factor 1 (somatomedin C)	*VCAN*	versican	*TNC*	tenascin C
*CHST11*	carbohydrate (chondroitin 4) sulphotransferase 11	*IGF2*	insulin-like growth factor 2 (somatomedin A)	*BMP6*	bone morphogenetic protein 6
*TIPARP*	TCDD-inducible poly(ADP-ribose) polymerase	*CDH11*	cadherin 11, type 2, OS-cadherin (osteoblast)	*ITGA11*	integrin, alpha 11
*IGF2*	insulin-like growth factor 2 (somatomedin A)	*TNC*	tenascin C	*GLI2*	GLI family zinc finger 2
*CDH11*	cadherin 11, type 2, OS-cadherin (osteoblast)	*BMP6*	bone morphogenetic protein 6	*LRRC17*	leucine rich repeat containing 17
*TGFBI*	transforming growth factor, beta-induced, 68 kDa	*ITGA11*	integrin, alpha 11	*CYP24A1*	cytochrome P450, family 24, subfamily A, polypeptide 1
*VDR*	vitamin D (1,25- dihydroxyvitamin D3) receptor	*GLI2*	GLI family zinc finger 2	*IGF2*	insulin-like growth factor 2 (somatomedin A)
*HHIP*	hedgehog interacting protein	*COL10A1*	collagen, type X, alpha 1	*RSPO2*	R-spondin 2
*EBP*	emopamil binding protein (sterol isomerase)	*LRRC17*	leucine rich repeat containing 17	*SMO*	smoothened, frizzled class receptor
*BMP6*	bone morphogenetic protein 6	*CYP24A1*	cytochrome P450, family 24, subfamily A, polypeptide 1	*HDAC5*	histone deacetylase 5
*GLI2*	GLI family zinc finger 2	*MAPK8*	mitogen-activated protein kinase 8	*WNT11*	wingless-type MMTV integration site family, member 11
*COL10A1*	collagen, type X, alpha 1	*TUFT1*	tuftelin 1	*DLX5*	distal-less homeobox 5
*HOXC9*	homeobox C9	*TEK*	TEK tyrosine kinase, endothelial	*BMP2*	bone morphogenetic protein 2
*LRRC17*	leucine rich repeat containing 17	*IGF2*	insulin-like growth factor 2 (somatomedin A)	*COL1A1*	collagen, type I, alpha 1
*DLX2*	distal-less homeobox 2	*RSPO2*	R-spondin 2	*LRRC17*	leucine rich repeat containing 17
*COL5A2*	collagen, type V, alpha 2	*SMO*	smoothened, frizzled class receptor	*CYP24A1*	cytochrome P450, family 24, subfamily A, polypeptide 1
*ADAMTS4*	ADAM metallopeptidase with thrombospondin type 1 motif, 4	*HDAC5*	histone deacetylase 5	*NOG*	noggin
*TEK*	TEK tyrosine kinase, endothelial	*PTGS2*	prostaglandin-endoperoxide synthase 2 (prostaglandin G/H synthase and cyclooxygenase)	*TMEM119*	transmembrane protein 119
*DLG1*	discs, large homolog 1 (Drosophila)	*WNT11*	wingless-type MMTV integration site family, member 11		
*IGF2*	insulin-like growth factor 2 (somatomedin A)	*GNAS*	GNAS complex locus		
*RSPO2*	R-spondin 2	*MMP14*	matrix metallopeptidase 14 (membrane-inserted)		
*EXTL1*	exostosin-like glycosyltransferase 1	*ASPN*	asporin		
*LEPRE1*	leucine proline-enriched proteoglycan (leprecan) 1	*CDH11*	cadherin 11, type 2, OS-cadherin (osteoblast)		
*SMO*	smoothened, frizzled class receptor	*DLX5*	distal-less homeobox 5		
*THBS1*	thrombospondin 1	*MMP2*	matrix metallopeptidase 2 (gelatinase A, 72 kDa gelatinase, 72 kDa type IV collagenase)		
*PBX1*	pre-B-cell leukaemia homeobox 1	*BMP2*	bone morphogenetic protein 2		
*WNT11*	wingless-type MMTV integration site family, member 11	*MAPK8*	mitogen-activated protein kinase 8		
*GNAS*	GNAS complex locus	*SLC26A2*	solute carrier family 26 (anion exchanger), member 2		
*TGFB2*	transforming growth factor, beta 2	*FOXS1*	forkhead box S1		
*SOX4*	SRY (sex determining region Y)-box 4	*COL1A1*	collagen, type I, alpha 1		
*COL3A1*	collagen, type III, alpha 1	*FSTL3*	follistatin-like 3 (secreted glycoprotein)		
*RUNX3*	runt-related transcription factor 3	*LRRC17*	leucine rich repeat containing 17		
*CSRNP1*	cysteine-serine-rich nuclear protein 1	*CYP24A1*	cytochrome P450, family 24, subfamily A, polypeptide 1		
*CDH11*	cadherin 11, type 2, OS-cadherin (osteoblast)	*SPARC*	secreted protein, acidic, cysteine-rich (osteonectin)		
*DLX5*	distal-less homeobox 5	*PDLIM7*	PDZ and LIM domain 7 (enigma)		
*MMP2*	matrix metallopeptidase 2 (gelatinase A, 72 kDa gelatinase, 72 kDa type IV collagenase)	*NOG*	noggin		
*BMP2*	bone morphogenetic protein 2	*TMEM119*	transmembrane protein 119		
*CYTL1*	cytokine-like 1				
*WNT7B*	wingless-type MMTV integration site family, member 7B				
*COL11 A1*	collagen, type XI, alpha 1				
*FOXS1*	forkhead box S1				
*COL1A1*	collagen, type I, alpha 1				
*SGPL1*	sphingosine-1-phosphate lyase 1				
*LRRC17*	leucine rich repeat containing 17				
*SUFU*	suppressor of fused homolog (Drosophila)				
*HES7*	hes family bHLH transcription factor 7				
*SPARC*	secreted protein, acidic, cysteine-rich (osteonectin)				
*NOG*	noggin				
*THBS1*	thrombospondin 1				
*MATN3*	matrilin 3				
*CADM1*	cell adhesion molecule 1				
*POSTN*	periostin, osteoblast specific factor				
*CADM1*	cell adhesion molecule 1				

### Molecular phenotype of TGF-β1-induces adipocytic cells

**Figure 4:**
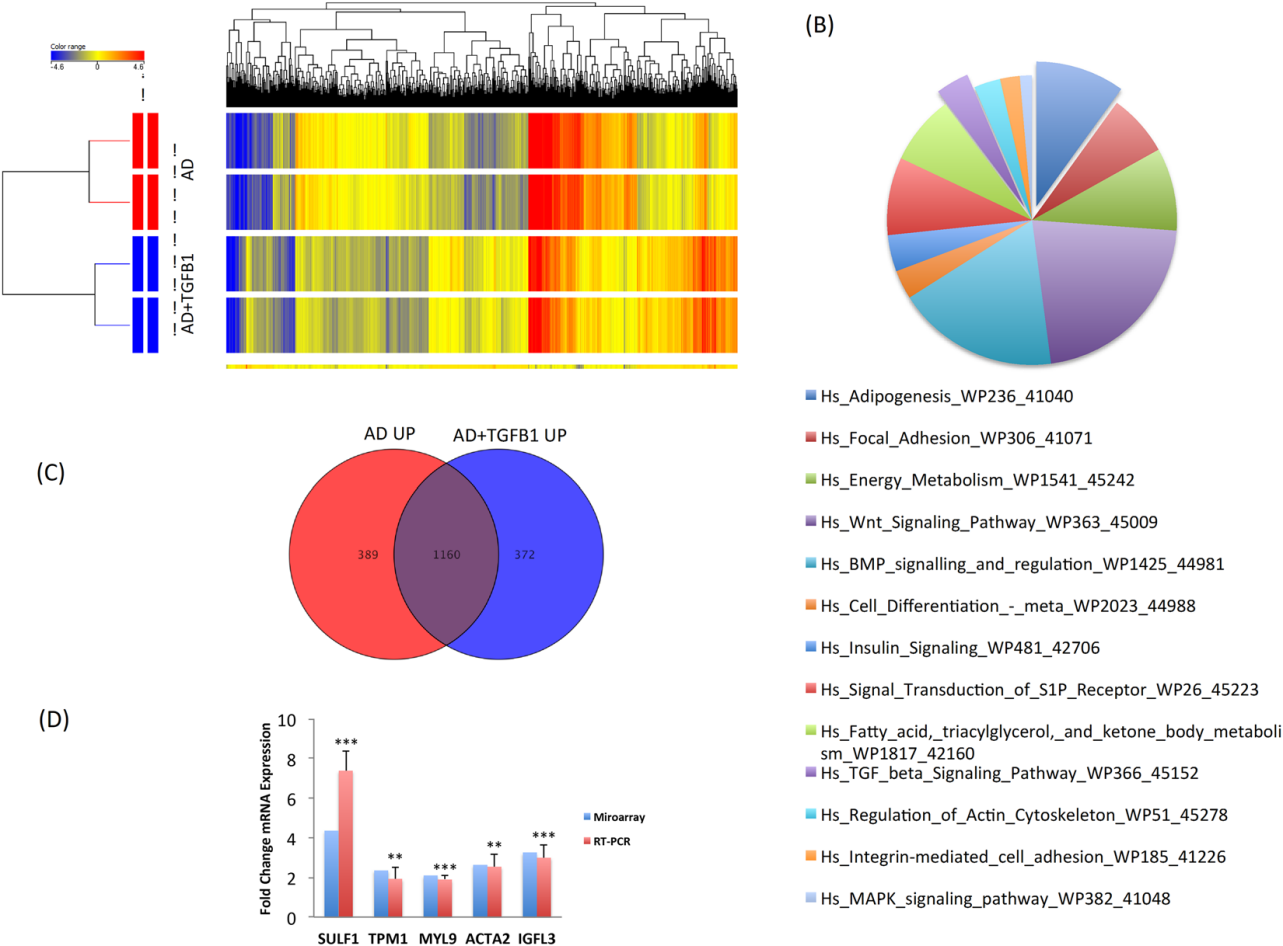
Gene expression profiling of hBMSC induced into adipocytes in the presence of TGF-β1. **(A)** Hierarchical clustering of human bone marrow stromal (skeletal) stem cells (hBMSC) induced into adipocytes (AD) (day 3) in the presence or absence of TGF-βl. Each row represents one replica sample and each column represents a transcript. Expression level of each gene in a single sample is depicted according to the colour scale. **(B)** Pie chart illustrating the distribution of the top 13 pathway designations for the up-regulated genes in TGF-β1 treated cells during adipogenesis. **(C)** Venn diagram depicting the overlap between the up-regulated genes during AD induction of hBMSC and the upregulated genes in hBMSC induced to AD in presence of TGF-β1. **(D)** qRT-PCR validation of selected genes from the microarray data (*SULF1*, *TPM1*, *MYL9*, *ACTA2*, and *IGFL3*).

Global gene expression profiling was conducted on cells treated with or without TGF-β1 (10 ng/ml) (day 3) in the presence of adipogenic induction medium. As shown in Fig. 4A, hierarchical clustering based on differentially expressed genes revealed clear separation of control samples treated with adipocyte induction medium alone (AD-differentiated) and those induced in presence of TGF-βl (AD-differentiated-TGF-β1). We identified 323 up- and 369 down-regulated genes in AD-differentiated-TGF-β1 compared to AD-differentiated cells (2.0 FC, p < 0.05; Supplementary Table S4, S5). Pathway analysis on the up-regulated genes in AD-differentiated-TGF-β1 treated samples revealed significant enrichment in several pathways related to adipogenesis, e.g. ‘adipogenesis’, ‘energy metabolism’, and ‘insulin signalling’ (Fig. 4B and Table 3). Comparing the list of the up-regulated genes in AD-differentiated cells and those in AD-differentiated-TGF-β1 cells revealed 1160 common genes that are predicted to be regulated by TGF-β signalling and also involved in AD-differentiation (Fig. 4C, Supplementary Table S6). The selectively up-regulated genes in AD-differentiated-TGF-β1 were enriched in categories of ‘fat cell differentiation’, ‘fatty acid derivative biosynthesis process’, ‘fatty acid derivative metabolic process’, and ‘inositol lipid-mediated signalling’ (Table 4). qRT-PCR findings showed concordance with microarray data for a selected gene panel of the up regulated genes in TGF-β1 treated cells (Fig. 4D).

**Table 3:** Up regulated genes involved in adipogenesis pathway in TGF-β1 treated cells after adipogenic induction.

Probe Name	Gene Name	Gene Symbol	FC ([AD + TGFB1] vs. [AD])
A_23_P53891	Kruppel-like factor 5 (intestinal)	*KLF5*	6.4302254
A_23_P13907	insulin-like growth factor 1 (somatomedin C)	*IGF1*	5.5686526
A_23_P46936	early growth response 2	*EGR2*	3.4646597
A_23_P18447	peroxisome proliferator-activated receptor gamma, coactivator 1 alpha	*PPARGC1A*	3.062086
A_24_P22079	forkhead box O1	*FOXO1*	2.6298373
A_33_P3302295	forkhead box C2 (MFH-1, mesenchyme forkhead 1)	*FOXC2*	2.3352392
A_23_P137381	inhibitor of DNA binding 3, dominant negative helix-loop-helix protein	*ID3*	2.3178163
A_33_P3237150	bone morphogenetic protein 2	*BMP2*	2.2145567
A_24_P38276	frizzled class receptor 1	*FZD1*	2.089059
A_24_P154037	insulin receptor substrate 2	*IRS2*	2.0130024
A_23_P211007	nuclear receptor interacting protein 1	*NRIP1*	2.0108652

**Table 4:** Up-regulated biological processes and related genes in TGF-β1 treated cells during adipogenesis using GO analysis.

Fat Cell Differentiation		Fatty Acid Derivative Biosynthesis Process		Fatty Acid Derivative Metabolic Process		Inositol Lipid-Mediated Signalling	
Gene Symbol	Gene Name	Gene Symbol	Gene Name	Gene Symbol	Gene Name	Gene Symbol	Gene Name
*RGS2*	regulator of G-protein signalling 2, 24 kDa	*EDN1*	endothelin 1	*EDN1*	endothelin 1	*IGF1*	insulin-like growth factor 1 (somatomedin C)
*INHBB*	inhibin, beta B	*PTGS1*	prostaglandin-endoperoxide synthase 1 (prostaglandin G/H synthase and cyclooxygenase)	*PTGS1*	prostaglandin-endoperoxide synthase 1 (prostaglandin G/H synthase and cyclooxygenase)	*FGF1*	fibroblast growth factor 1 (acidic)
*PPARGC1A*	peroxisome proliferator-activated receptor gamma, coactivator 1 alpha	*PTGS2*	prostaglandin-endoperoxide synthase 2 (prostaglandin G/H synthase and cyclooxygenase)	*ALOX15B*	arachidonate 15-lipoxygenase, type B	*EDN1*	endothelin 1
*RUNX1T1*	runt-related transcription factor 1; translocated to, 1 (cyclin D-related)	*PTGS1*	prostaglandin-endoperoxide synthase 1 (prostaglandin G/H synthase and cyclooxygenase)	*PTGS2*	prostaglandin-endoperoxide synthase 2 (prostaglandin G/H synthase and cyclooxygenase)	*NPR3*	natriuretic peptide receptor 3
*EGR2*	early growth response 2	*GGT5*	gamma-glutamyltransferase 5	*PTGS1*	prostaglandin-endoperoxide synthase 1 (prostaglandin G/H synthase and cyclooxygenase)	*NRG1*	neuregulin 1
*FOXO1*	forkhead box O1	*C9orf3*	chromosome 9 open reading frame 3	*GGT5*	gamma-glutamyltransferase 5	*IRS2*	insulin receptor substrate 2
*PTGS2*	prostaglandin-endoperoxide synthase 2 (prostaglandin G/H synthase and cyclooxygenase)			*C9orf3*	chromosome 9 open reading frame 3	*FOXO1*	forkhead box O1
						*FGF7*	fibroblast growth factor 7

### Identification of *SERPINB2* as a TGF-β-responsive gene

**Figure 5:**
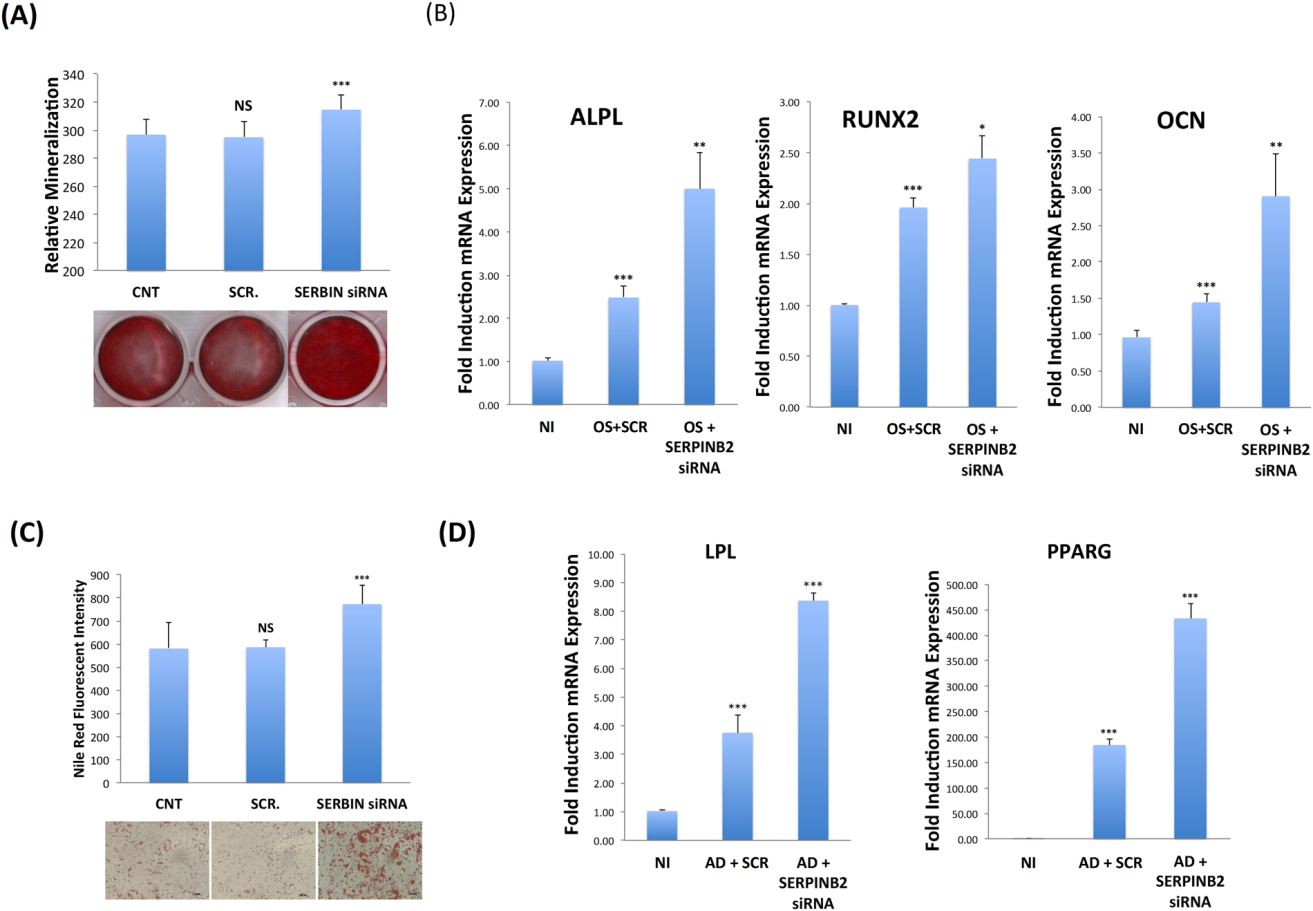
*SERPINB2* is a TGF-β-target gene that is suppressed in human bone marrow stromal (skeletal) stem cells (hBMSC) during osteoblast and adipocyte differentiation. (**A**) qRT-PCR performed for TGF-β responsive genes: *SERPINB2, ACTA2*, and *TPM2* for controls (CNT), as well as for cells treated with: SB-431542 (SB) and TGF-β1. Expression of each target gene was normalised to *GAPDH*. Data are shown as the SD of three independent experiments (**B**) qRT-PCR showing time course of *SERPINB2* expression between day (**D**) 0, D1, D3, D5, and D7 for cells induced with either osteoblast (OS) or adipocyte (AD) induction medium. (**C**) qRT-PCR of *SERPINB2* mRNA expression on day 3 post transfection with SERPINB2-specific or scrambled control siRNA. Data are presented as fold change mRNA expression ± SD from three independent experiments (**D**) Western blot analysis of SERPINB2 in SERPINB2-siRNA-depleted cells compared to scramble-siRNA transfected control cells (upper panel). B-Actin (ACTB, lower panel) was used as a loading control. (**E**) Cell viability measured using Alamar blue assay for hBMSCs on days 2, 4, 6, and 8 post transfection with scrambled (SCR) or SERPINB2-specific siRNA. (**F**) qRT-PCR for *ACTA2*, and *TPM1* expression on day 3 post transfection with SERPINB2-specific or scramble (SCR) siRNA, in the presence or absence of SB-431542. Data are presented as mean fold change in mRNA expression ± SD, from three independent experiments. (**G**) Western blot analysis for P-c-JUN and P-JNK in SERPINB2-depleted cells compared to scramble transfected control cells (upper panel), whereas B-Actin (ACTB, lower panel) was used as a loading control. ***p < 0.005.

Among the genes highly regulated by TGF-β1 treatment, we identified *SERPINB2* (significantly down-regulated), *ACTA2* (6 fold up-regulated), and *TPM1* (4 fold up-regulated) (Fig. 5A). The specificity of TGF-β1 induction of *SERPINB2* was demonstrated by SB-431542 treatment, which exerted an opposite effect (Fig. 5A). As shown in Fig. 5B, the enhanced OS and AD differentiation was associated with reduced expression of *SERPINB2* during the course of differentiation, which reached a minimum (close to zero) during AD differentiation. Further, we interestingly observed by gene expression analysis that SERPINB2 gene is negatively regulated by TGF-β1 and positively upon inhibition of TGF-β1 pathway by SB, and this finding was consistent both during osteoblast and adipocyte differentiation (Fig. s8)

### Silencing of SERPINB2 in hBMSC promotes OS and AD differentiation

As osteoblastic and adipocytic differentiation was associated with reduced *SERPINB2* expression, we employed a loss-of-function approach to assess the specific role of SERPINB2 in the differentiation processes. siRNA targeting of *SERPINB2* successfully down-regulated *SERPINB2* gene expression (Fig. 5C) and protein expression as demonstrated by qRT-PCR and Western blot (Fig. 5D), respectively. No significant changes were observed in cell proliferation in SERPINB2-depleted cells as compared to siRNA-treated controls (Fig. 5E). The gene expression of *ACTA2* and *TPM1*, which are TGF-β responsive genes, was up-regulated in SERPINB2-depleted cells (Fig. 5F). Phosphorylated c-Jun and phosphorylated JNK proteins, which are modulators of TGF-β signalling pathways, were found to be induced in SERPINB2-depleted cells (Fig. 5G).

**Figure 6:**
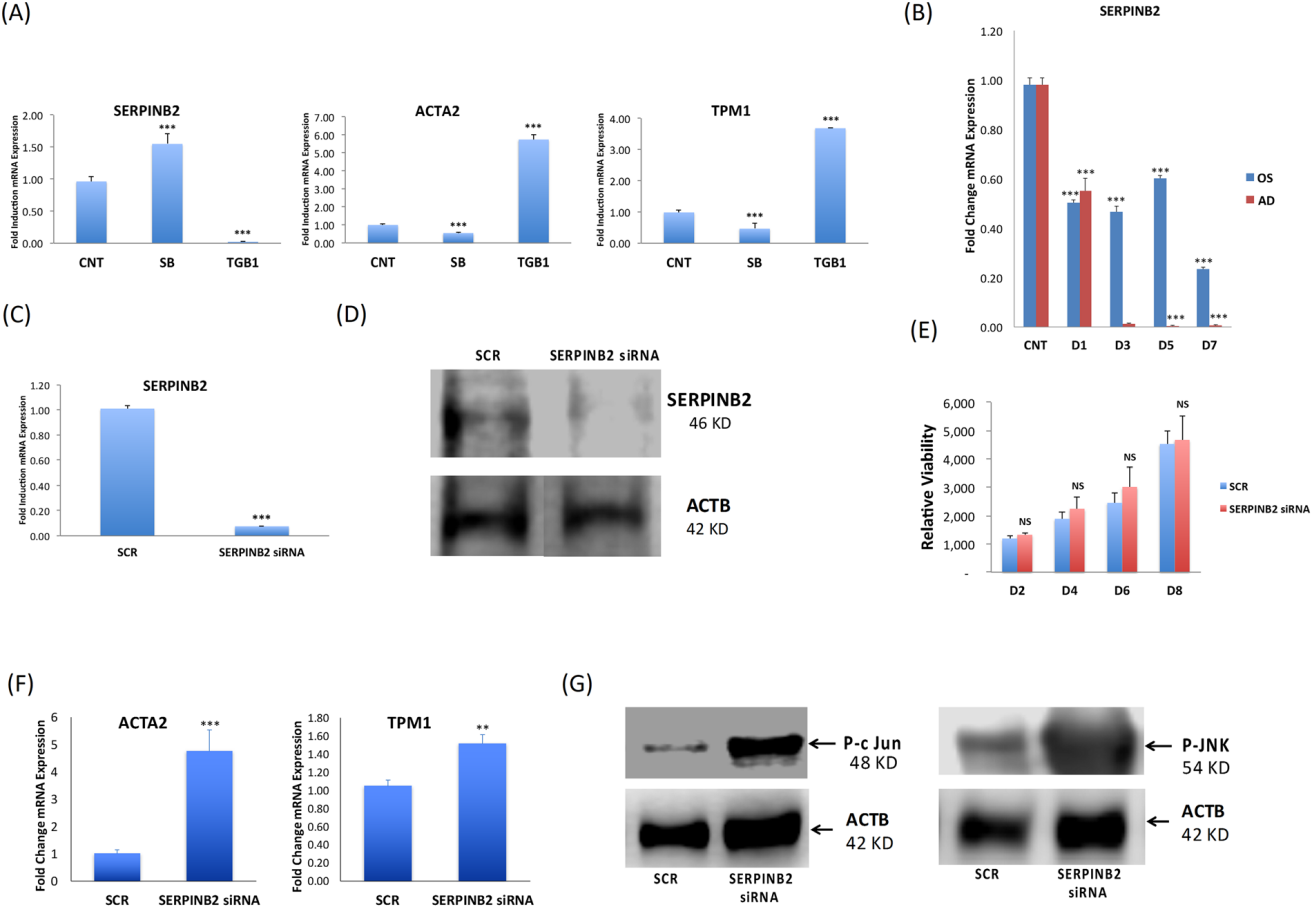
Down-regulation of SERPINB2 promotes osteoblastic and adipocytic differentiation of human bone marrow stromal (skeletal) stem cells (hBMSC) **(A)** mineralised matrix deposition was assessed using Alizarin Red S staining (lower panel). Quantification of mineralised matrix formation under control (CNT), cells transfected with either transfection with SERPINB2-specific (SERBIN siRNA) or scramble (SCR) siRNA (upper panel). Data are presented as relative mineralisation ± SD from three independent experiments. **(B)** qRT-PCR quantification of the osteoblastic markers (ALPL, RUNX2, and OCN) on day 7 under the indicated treatment conditions, OS (osteoblast differentiation). Expression of each target gene was normalised to GAPDH. Data are presented as the means ± SD from three independent experiments, hBMSCs under different experimental conditions were induced into adipocytes for 5 days and subsequently stained using Oil Red O **(C**, lower panel). Data are shown as microscopic images (40×). Nile red stain quantification is shown in the upper panel. Data are presented as mean relative Nile red staining intensity ± SD from three independent experiments. **(D)** qRT-PCR quantification for the adipogenic markers (PPARG2 and LPL) for hBMSCs under different treatment conditions. AD (adipocyte differentiation). Expression of each target gene was normalised to GAPDH. Data are presented as fold change in mRNA expression ± SD from three independent experiments. *p < 0.05; **p < 0.005, ***p < 0.0005.

Notably, siRNA-mediated SERPINB2 inhibition led to enhanced OS and AD differentiation as evident by increased mineralised matrix formation (Fig. 6D), up-regulation of specific osteoblastic markers: ALPL, RUNX2, and OCN (Fig. 6E), increased numbers of mature adipocytes (Fig. 6F), and up-regulation of the adipogenic gene markers *LPL* and *PPARG2* (Fig. 6G). These effects on hBMSCs differentiation were further confirmed by using primary human bone-marrow derived MSC, where similar results were obtained (Fig. s9).

### SB-431542 rescued SERPINB2-siRNA-induced enhanced differentiation hMSC

**Figure 7:**
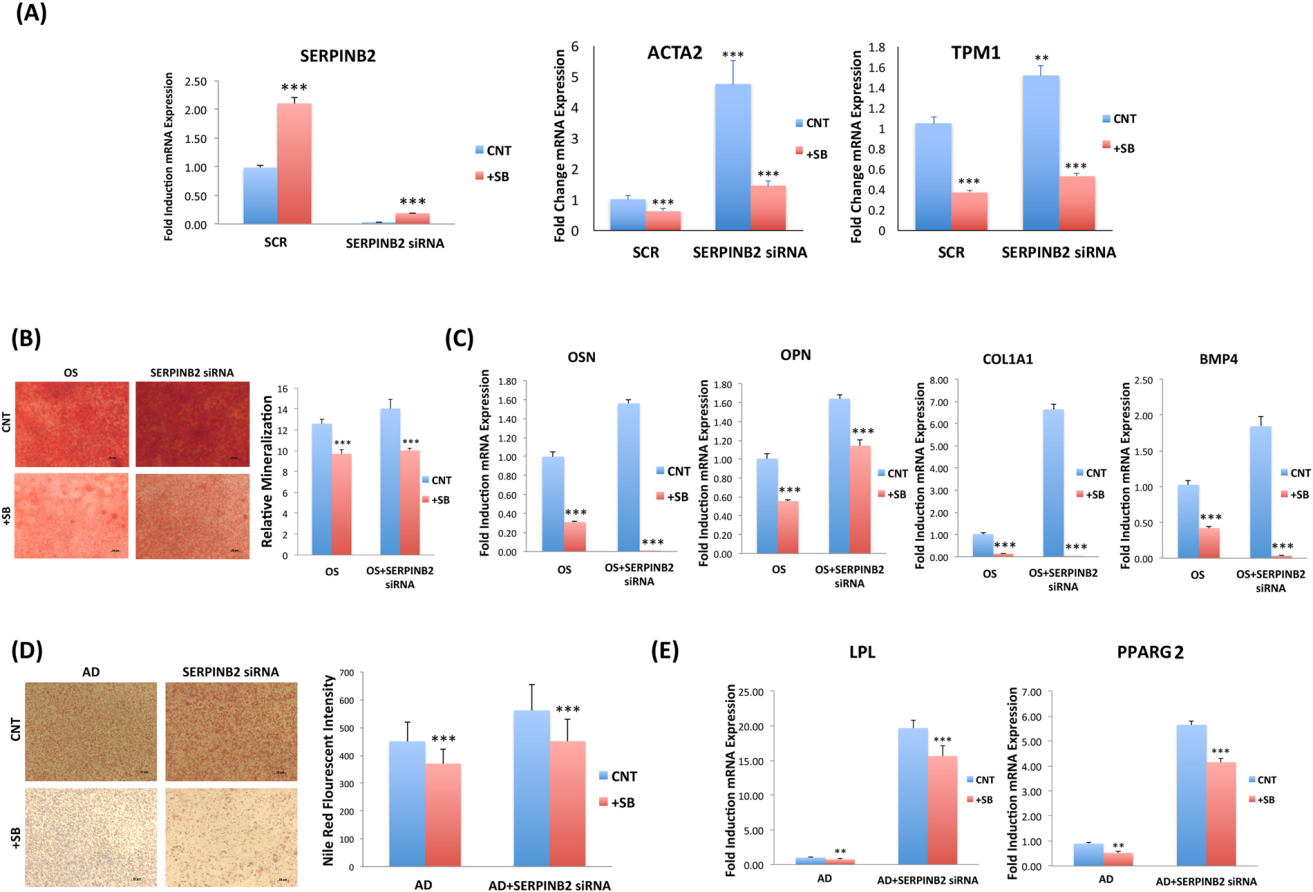
Inhibition of TGFB signaling reversed the enhanced osteoblastic and adipocytic differentiation observed in SERPINB2-depleted human bone marrow stromal (skeletal) stem cells (hBMSC). (**A**) q-RT-PCR for SERPINB2, ACTA2, and TPM1 expression on day 3 post transfection with SERPINB2-specific-siRNA or Scramble siRNA (SCR), in the presence or absence of SB-431542 (SB). Data are presented as mean fold change in mRNA expression ± SD, from three independent experiments. (**B**) Mineralized matrix deposition was determined by Alizarin Red S staining presented as microscopic images (magnification 40X) of stained wells (left panel). Quantification of mineralized matrix formation under different treatment conditions is presented in the right panel, OS (osteoblast differentiation). Data are presented as mean relative mineralization ± SD from three independent experiments; (**C**) qRT-PCR for OSN, OSP, COL1A1 and BMP4 osteoblastic markers. Expression of each target gene was normalized to GAPDH. Data are presented as mean ± SD from three independent experiments. Cells were induced into adipocyte (AD) for 5 days in the presence or absence of SB-431542 (SB) and stained using Oil Red O (**D**) as shown in the representative microscopic images (left panel) as well as Nile Red stain quantification (right panel) under different experimental conditions: AD induced of scramble-siRNA (CNT), and adipo-induced SERPINB2-siRNA-depleted cells. (**E**) qRT-PCR quantification for PPARG2, and LPL adipocytic markers under the indicated treatments. Expression of each target gene was normalized to GAPDH. Data are presented as mean ± SD from three independent experiments. n = 6. **p < 0.005, ***P < 0.0005.

To investigate the effect of induced expression of SERPINB2 on hBMSC differentiation, we conducted rescue experiment using SB-431542 to induce the endogenous SERPINB2. Treating SERPINB2-defecient cells with SB-431542 significantly upregulated *SERPINB2* gene expression (>6.0 FC; Fig. 7A). The gene expression of *ACTA2* and *TPM1*, which are TGFB responsive genes, were up-regulated in SERPINB2-depleted cells. Enhanced OS and AD differentiation in SERPINB2-depleted cells were significantly reversed in the presence of SB-431542 as shown by reduced mineralized matrix formation (Fig. 7B) and down-regulation of osteoblastic gene markers: *OCN, OPN, COL1a1*, and *BMP4* (Fig. 7C), and reduced number of mature adipocytes (Fig. 7D) as well as down-regulation of adipocytic gene markers: *LPL* and *PPARG2* (Fig. 7E).

### Molecular phenotype of SERPINB2-depleted hBMSCs

**Figure 8:**
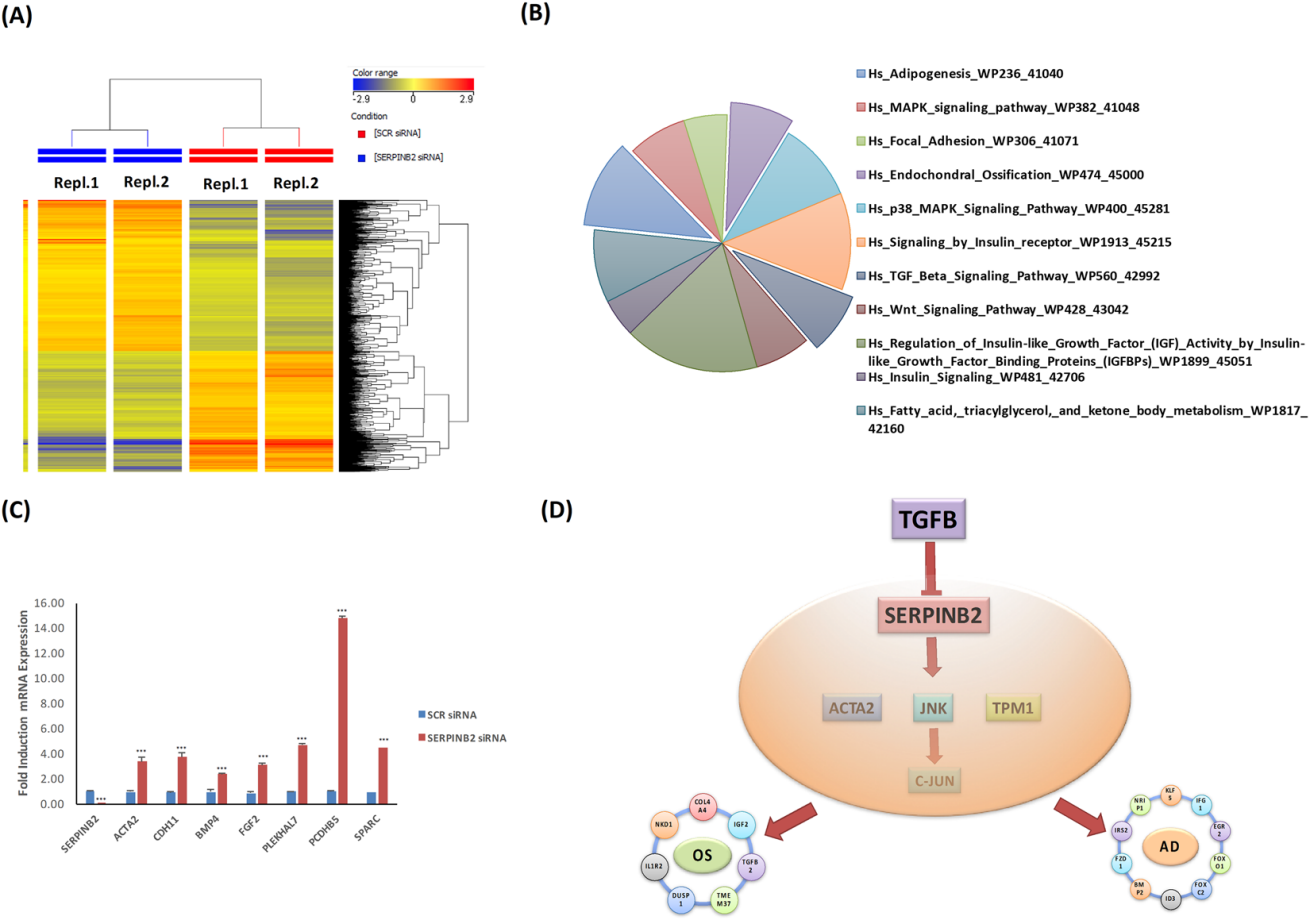
Gene expression profiling of SERPINB2-depleted human bone marrow stromal (skeletal) stem cells (hBMSC). (**A**) Hierarchical clustering of SERPINB2-depleted hBMSCs compared to scramble transfected control cells, based on differentially expressed mRNA transcripts. Each column represents one replica sample and each row represents a transcript. Expression level of each gene in a single sample is depicted according to the colour scale. **(B)** Pie chart illustrating the distribution of 11 pathways out of the top pathway designations for the de-regulated genes in SERPINB2-depleted hBMSCs. **(C)** The expression levels of selected genes from the microarray data were validated using qRT-PCR in SERPINB2-depleted hBMSC. Data are presented as the means ± SD from two independent experiments, *n* = 6 **p < 0.005; ***p < 0.0005. Scrambled cells were used as a control. **(D)** Proposed working model illustrating the biological role for TGF-β1 in promoting osteogenesis and adipogenesis through suppression of SERPINB2 and possible down-stream targets.

Global gene expression profiling was conducted on SERPINB2-depleted hBMSCs and compared with control scramble siRNA transfected cells. Hierarchical clustering based on differentially expressed genes revealed clear separation of control cells from SERPINB2-siRNA transfected cells (Fig. 8A). We identified 836 up-regulated and 670 down-regulated genes in SERPINB2-depleted cells compared to control cells (2.0 FC, p < 0.05; Supplementary Table S7). Pathway analysis performed on the up-regulated genes revealed significant enrichment in several genetic pathways related to ‘endochondral ossification’, ‘adipogenesis’, ‘TGF-β signalling’, ‘WNT signalling’, and ‘MAPK signalling’ pathways (Fig. 8B). Good concordance was observed between microarray and qRT-PCR results for a selected regulated gene panel (Fig. 8C).

## Discussion

The TGFβ signalling pathway plays a regulatory role in hBMSC biology; however, the molecular details of its action have not been elucidated. In the present study, we investigated the role of TGF-β1 during OS and AD differentiation of hBMSC and described a novel TGF-β1-responsive gene in hBMSC: *SERPINB2*.

We observed differences in the biological effects of TGF-β1 on hBMSC differentiation dependent on the temporal exposure of the cells. Continuous treatment with TGF-β1 was more effective in enhancing OS differentiation, whereas a single treatment with TGF-β1 during the commitment phase preferentially enhanced AD differentiation. However, these effects were quantitative rather than qualitative and suggest that TGF-β1 is important for both OS and AD differentiation. Moreover, the specificity of the effects was demonstrated by using the TGF-β receptor kinase inhibitor SB-431542.

We observed that TGF-β1 promoted OS differentiation and up-regulation of osteoblastic genes (ALPL, RUNX2, and OSC). Previous studies have also demonstrated that TGF-β1 regulates the differentiation of osteo-progenitor cells^7–10^, although the direction of such regulation has been disputed. For example, some studies reported the inhibition of OS differentiation *in vitro* by TGF-β1^8, 11^. In contrast, it has also been reported that TGF-β1 stimulates bone matrix deposition and bone cell replication^12^, whereas inhibition of TGF-β1 signalling was shown to suppress OS differentiation^13^. Despite these contradictory data, most studies support a model in which TGF-β1 promotes bone formation by recruiting OS progenitors and inducing the formation of bone matrix at early stages of OS differentiation. However, at later stages of OS differentiation, TGF-β1 appears to inhibit OS differentiation and mineralisation^11, 14, 15^, probably secondary to regulation by other growth factors e.g. bone morphogenic proteins (BMPs)^16, 17^. The reported findings of TGF-β1 enhancing the expression of RUNX2 in combination with BMPs during the early stage of OS differentiation^18^ and its induction of SMAD2 at later stages of OS differentiation that, interacts with RUNX2 causing suppression of the expression of RUNX2 itself and of a consequently other osteoblastic genes including ALPL, collagen type 1, and oxidosqualene cyclase (OSC) by an auto-regulatory feedback mechanism^15, 19–23^, may explain this dual role of TGF-β1 in OS differentiation. These findings suggest that TGF-β1 effects are dependent on the differentiation stage of the target cells and/or presence of other interacting factors.

Although many studies have reported inhibitory effect of TGF-β1 on adipogenesis, some reports have demonstrated a pro-adipogenic effect of TGF-β1^24, 25^, which is more consistent with the observed TGF-β1 enhancement of bone marrow adipocyte formation observed in the current study. For example, the treatment of mouse NIH/3T3-L1 fibroblasts with TGF-β1 significantly suppressed mature adipocyte formation, irrespective of whether the treatment constituted a single dose at the initiation of differentiation or a continuous exposure^26^. Continuous exposure to TGF-β1 during adipogenic induction, inhibited adipogenic differentiation of mesenchymal cells, through repression of the function of the key adipogenic transcription factors C/EBPβ and C/EBPδ^27^. Furthermore, it was demonstrated that continuous exposure to TGF-β inhibited adipogenesis due to significant reduction in TGF-β receptor levels, which was mediated through SMAD2 and SMAD3^28^. In contrast, an early study of brown adipocytes in rat showed up-regulation of the expression of lipogenic enzymes upon treatment with 100 pM TGF-β1^25^. TGF-β1-induced inhibition models used either a low single dose (approximately 2 ng/ml) at the time of differentiation induction or a continuous dose (ranging from 0.2–10 ng/ml). Therefore, the discrepancies observed in different studies might reflect different cell types employed and experimental setups.

Among the genes highly regulated by TGF-β1 treatment, we identified *SERPINB2* as a novel gene supressed by TGF-β induction in hBMSC. The physiological role of SERPINB2 in bone biology has not previously been investigated. Plasminogen activator inhibitor-2 (SERPINB2), also called PAI-2, is a member of the Ov-serpin family of serine protease inhibitors^29^. It is expressed in particular in monocytes/macrophages, as well as in a wide range of other hematopoietic and non-hematopoietic cells^30^. SERPINB2 has been shown to bind several intracellular and extracellular proteins, indicating physiological function in both intracellular and extracellular compartments^31^. *SERPINB2* functions as a coagulation factor that inactivates urokinase and as a tissue-type plasminogen activator in the extracellular space and on the cell surface^29, 32^. *SERPINB2* is highly expressed in pregnancy, infection and inflammation^32^. Interestingly, SERPINB2 was downregulated in co-cultured hMSC with U87-MG (glioblastoma multiformae (GBM) cell line) suggesting a role in stroma-cancer cell interaction that needs to be clarified^33^. We have clearly shown that SERPINB2 is up-regulated when TGF-βpathway is blocked by SB which lead to inhibition of differention hBMSCs and the opposite was seen when we stimulated TGF-β pathway see Fig. 7.

In conclusion, our results provide additional details to the role of TGF-β intracellular signalling pathway in OS and AD differentiation of hBMSC and identify *SERPINB2 as* a negative regulator of these effects Fig. 8D illustrates our current working model for the role of TGF-β on hBMSC through the suppression of *SERPINB2* gene and we provide a possible list of down-stream targets *ACTA2*, *TPM1*, *c*-*JUN*, and *JNK*. Future studies are required to clarify the mechanistic role of SERPINB2 *in vivo* skeletal biology. SERPINB2 may also serve as a target to develop strategies for regulating bone mass and bone marrow fat.

## Methods

### Cell Culture

As a model for primary human bone marrow MSCs, the hMSC-TERT cell line was established from normal human bone marrow MSCs though overexpression of the human telomerase reverse transcriptase gene (*hTERT*)^34^. hMSC-TERT cells have been extensively characterised as exhibiting a similar cellular and molecular phenotype to primary hBMSCs^33^. In the current study, we employed a sub-clone derived from hMSC-TERT termed hMSC-TERT-CL1, which has been characterised previously^35^. For simplicity, we refer to this line as hBMSC throughout the manuscript.

The cells were cultured in Dulbecco’s modified Eagle medium (DMEM) supplemented with 4500 mg/l D-glucose, 4 mM L-glutamine, 110 mg/l sodium pyruvate, 10% foetal bovine serum (FBS), 1× penicillin-streptomycin (Pen-strep), and non-essential amino acids (all purchased from Gibco-Invitrogen, Carlsbad, CA).

### *In vitro* OS differentiation

Cells were grown in standard DMEM growth medium in 6-well plates at 0.3 × 10^6^ cells/ml. When 70–80% confluence was reached, test cells were cultured in DMEM supplemented with OS induction mixture containing 10% FBS, 1% Pen-strep, 50 μg/ml L-ascorbic acid (Wako Chemicals, Neuss, Germany), 10 mM β-glycerophosphate (Sigma, St. Louis, MO), and 10 nM calcitriol (1α, 25-dihydroxyvitamin D3; Sigma), and 10 nM dexamethasone (Sigma). The media were replaced 3 times per week. Cells cultured in standard culture medium were considered as the negative control.

For dose response experiments, cells were seeded and treated at D -2 with 10 ng/ml TGF-β1 for 2 days commitment stage, then differentiation stage was initiated by adding osteoblastic induction medium at D0, while TGF-β1 treatment was continued till day 7 in TGF-β -continuous treated cells.

### *In vitro* AD differentiation

Cells were grown in standard DMEM growth medium in 6-well plates at 0.3 × 10^6^ cells/ml. At 90–100% confluence, cells were switched to DMEM supplemented with adipogenic induction mixture containing 10% FBS, 10% horse serum (Sigma), 1% Pen-strep, 100 nM dexamethasone, 0.45 mM isobutyl methyl xanthine^36^ (Sigma)], 3 μg/ml insulin (Sigma), and 1 μM rosiglitazone^37^ (Novo Nordisk, Bagsvaerd, Denmark). The media were replaced 3 times per week. Cells cultured in standard culture medium were considered as the negative control. For dose response experiments, cells were seeded and treated at D -2 with 10 ng/ml TGF-β1 for 2 days commitment stage, then differentiation stage was initiated by adding adipocytic induction medium at D0, while TGF-β1 treatment was continued till day 7 in TGF-β -continuous treated cells.

### Cytochemical staining

#### Alizarin Red S staining for mineralised matrix

The cell layer was washed with phosphate buffered saline (PBS) and then fixed with 4% paraformaldehyde for 15 min at room temperature. After removing the fixative, the cell layer was rinsed in distilled water 3 times and stained using a 2% Alizarin Red S Staining Kit (Cat. No. 0223, ScienCell, Research Laboratories, Carlsbad, CA) for 20–30 min at room temperature. Excess dye was washed off with water 3–5 times; subsequently, the stained cells were kept in water to prevent drying out. Images were acquired using an inverted Zeiss microscope (Thornwood, NY). For quantification of Alizarin Red S staining, plates were air-dried and then the Alizarin Red S dye was eluted in 800 µl acetic acid incubated in each well for 30 min at room temperature as previously described^38^ and measured using an Epoch spectrophotometer (Bio-Tek, Winooski, VT) at 405 nm.

#### OsteoImage mineralisation assay

The *in vitro*-formed mineralised matrix was quantified using the OsteoImage Mineralization Assay Kit (Cat. No. PA-1503, LONZA, Allendale, NJ). Culture medium was removed and cells were washed once with PBS, then fixed with 70% cold ethanol for 20 min. An appropriate amount as recommended by the manufacturer of diluted staining reagent was added and plates were incubated in the dark for 30 min at room temperature. Cells were washed and staining intensity was assessed using a fluorescent plate reader at 492/520 excitation emission wavelengths.

#### Oil red-O staining for lipid droplets

Mature adipocytes filled with cytoplasmic lipid droplets were visualised by staining with Oil Red-O. After washing with PBS, the cells were fixed in 4% formaldehyde for 10 min at room temperature, then rinsed once with 3% isopropanol and stained for 1 h at room temperature using filtered Oil Red-O staining solution (prepared by dissolving 0.5 g Oil red-O powder in 60% isopropanol). To quantify the mature adipocytes formed, Oil Red O stain was eluted by adding 100% isopropanol to each well and absorbance was measured using an Epoch spectrophotometer at 510 nm.

#### Nile red fluorescence determination and quantification of mature adipocytes

Stock solution of Nile red (1 mg/ml) in DMSO was prepared and stored at −20 °C protected from light. Staining was performed after fixing the cells using 4% paraformaldehyde (Sigma) for 15 min at room temperature, then washed with PBS. The dye was added directly to the cells (5 μg/ml in PBS) and the cells were incubated for 10 min at room temperature. Fluorescent signal was measured with a SpectraMax/M5 fluorescence spectrophotometer plate reader (Molecular Devices Co., Sunnyvale, CA) using the bottom well-scan mode where nine readings were taken per well using excitation 485 nm and emission 572 nm spectra.

### Real time qRT-PCR

Total RNA was extracted from differentiating cells either treated or not treated (control samples) with single dose of TGF-β1 at D5 of induction using a PureLink RNA mini isolation kit (Cat No: 12183018 A, Ambion by Life Technologies, Austin, TX) as recommended by the manufacturer. Total RNA was quantified using a Nanodrop spectrophotometer (Nanodrop 2000, Thermo Scientific, Waltham, MA). cDNA was synthesised from 1 μg of the RNA samples using a High Capacity cDNA Reverse Transcription kit (Applied Biosystems, Foster City, CA) using a Labnet, Multigene thermocycler (Edison, NJ) according to the manufacturer’s instructions. Relative levels of mRNA were determined from cDNA using real time PCR (Applied Biosystem-Real Time PCR Detection System) with a Power SYBR Green PCR kit (Applied Biosystems, UK), or with the TaqMan Universal master Mix II, no UNG (Applied Biosystems, Foster City, CA) according to the manufacturer’s instructions. Following normalisation to the reference gene *GAPDH*, quantification of gene expression was carried out using a comparative Ct method where ΔCT is the difference between the CT values of the target and reference gene. Primers (Supplementary Tables S8 and S9) were either obtained directly from Applied Biosystems (Foster City, CA) as TaqMan primers or were synthesised by Life Technologies based on previously published primer sequences.

### DNA microarray global gene expression profiling

Total RNA was extracted from differentiating cells either treated or not treated (control samples) with single dose of TGF-β1 at D5 of induction using the PureLink RNA mini isolation kit as recommended by the manufacturer. Then, 150 ng total RNA was labelled and hybridised to the Agilent Human SurePrint G3 Human GE 8 × 60 k microarray chip (Agilent Technologies, Santa Clara, CA). All microarray experiments were conducted at the Microarray Core Facility (Stem Cell Unit, King Saud University College of Medicine). Normalisation and data analyses were conducted using GeneSpring GX software (Agilent Technologies). Pathway analysis was conducted using the Single Experiment Pathway analysis feature in GeneSpring 12.0 (Agilent Technologies). A two-fold cut off with p < 0.05 was used.

### Western blotting

Whole cell lysates were prepared as previously described^38^. Soluble proteins were analysed by immunoblotting with antibodies anti-SERPINB2, anti-P-c Jun, and anti-P-UNK (ThermoFisher Scientific, diluted 1:5000), and anti-β-ACTIN (Sigma, A3854, diluted 1:10,000). Reactivity was detected with horseradish peroxidase-conjugated secondary antibodies (Santa-Cruz Biotechnology, Dallas, TX) and Clarity western ECL substrate (Bio-Rad Laboratories, Berkeley, CA) for chemiluminescence using a C-Digit Blot Scanner (LI-COR, Lincoln, NE).

### siRNA transfection

For transfection, hBMSC cells in logarithmic growth phase were reverse-transfected with Silencer Select Pre-designed SERPINB2-siRNA (25 nM) (Ambion ID: s10016, s10017, and s10018, Cat. no. 4392420, Thermo Fisher Scientific Life Sciences) using Lipofectamine RNAiMAX Reagent (Invitrogen) plus serum-free Opti-MEM® I medium (Thermo Fisher Scientific Life Sciences) as per the manufacturer recommendation. On day 3 of transfection, the cells were induced into OSs or ADs for an additional 5 days.

### Statistical analysis

All results presented are given as the means ± standard deviation (SD) of at least 3 independent experiments, unless indicated otherwise. A student’s t-test was used for testing differences between groups. p-values < 0.05 were considered statistically significant.

## Electronic supplementary material

Supplementary information

Table s1

Table s2

Table s3

Table s4

Table s5

Table s6

Table s7
