# Supplementary material for: SERPINB2 is a novel TGFβ-responsive lineage fate determinant of human bone marrow stromal cells
Source: Sci Rep. 2017 Sep 7;7:10797. doi: 10.1038/s41598-017-10983-x (PMC5589808; doi:10.1038/s41598-017-10983-x)
Supplement: Supplementary file 1 — Supplementary information [file 41598_2017_10983_MOESM1_ESM.pdf]

SERPINB2 is a novel TGFβ-responsive lineage fate determinant of human bone marrow stromal cells

Mona Elsafadi[1,2]*, Muthurangan Manikandan[1], Muhammad Atteya[1,3], Raed Abu Dawud[4,5], Sami Almalki[6], Zahid Ali Kaimkhani[1], Abdullah Aldahmash[1,7], Nehad M Alajez[1], Musaad Alfayez[1], Moustapha Kassem[1,2], & Amer Mahmood[1]*

Supplementary Figure s1.

Pathway analysis of the up-regulated genes revealed significant enrichment in several pathways related to ‘endochondral ossification’ pathway.

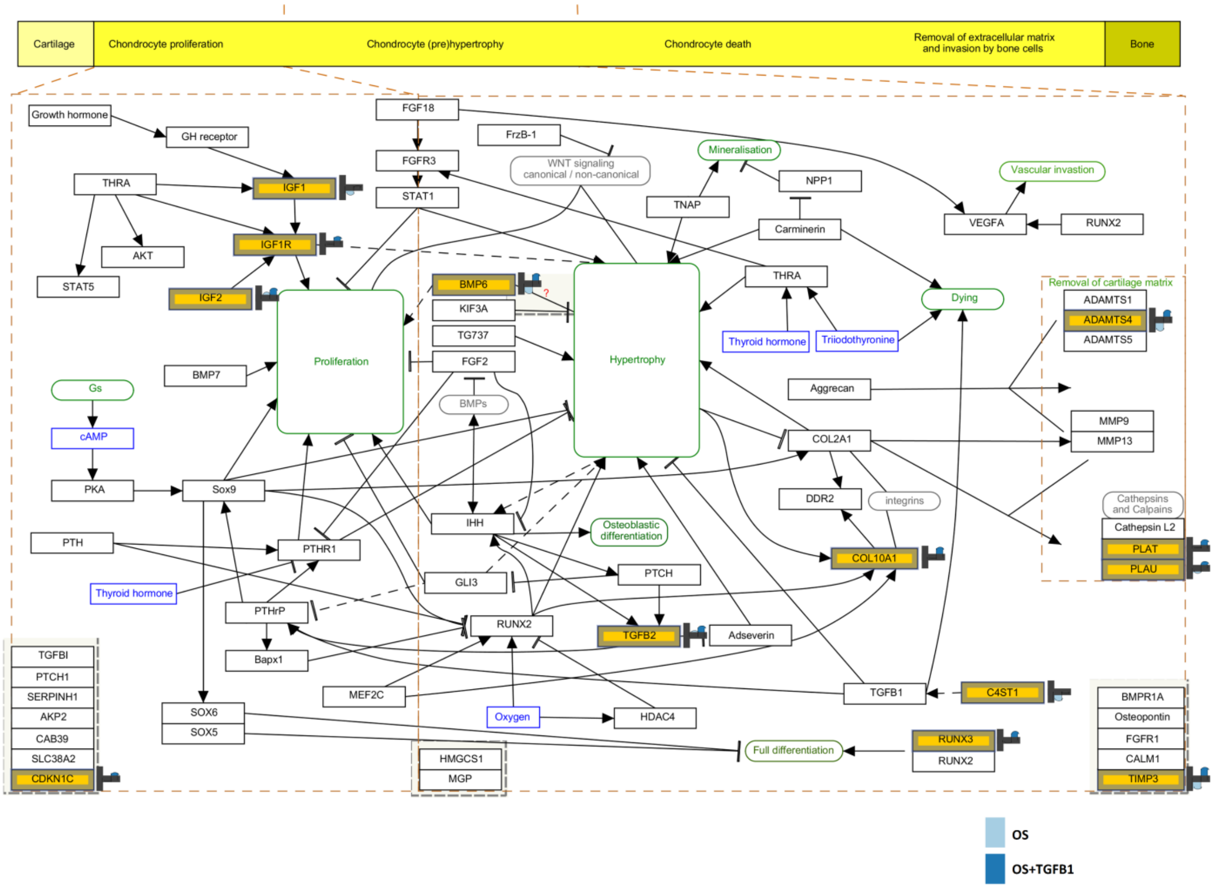

Supplementary Figure s2.

Pathway analysis of the up-regulated genes revealed significant enrichment in several pathways related to ‘matrix metalloproteinases’ pathways.

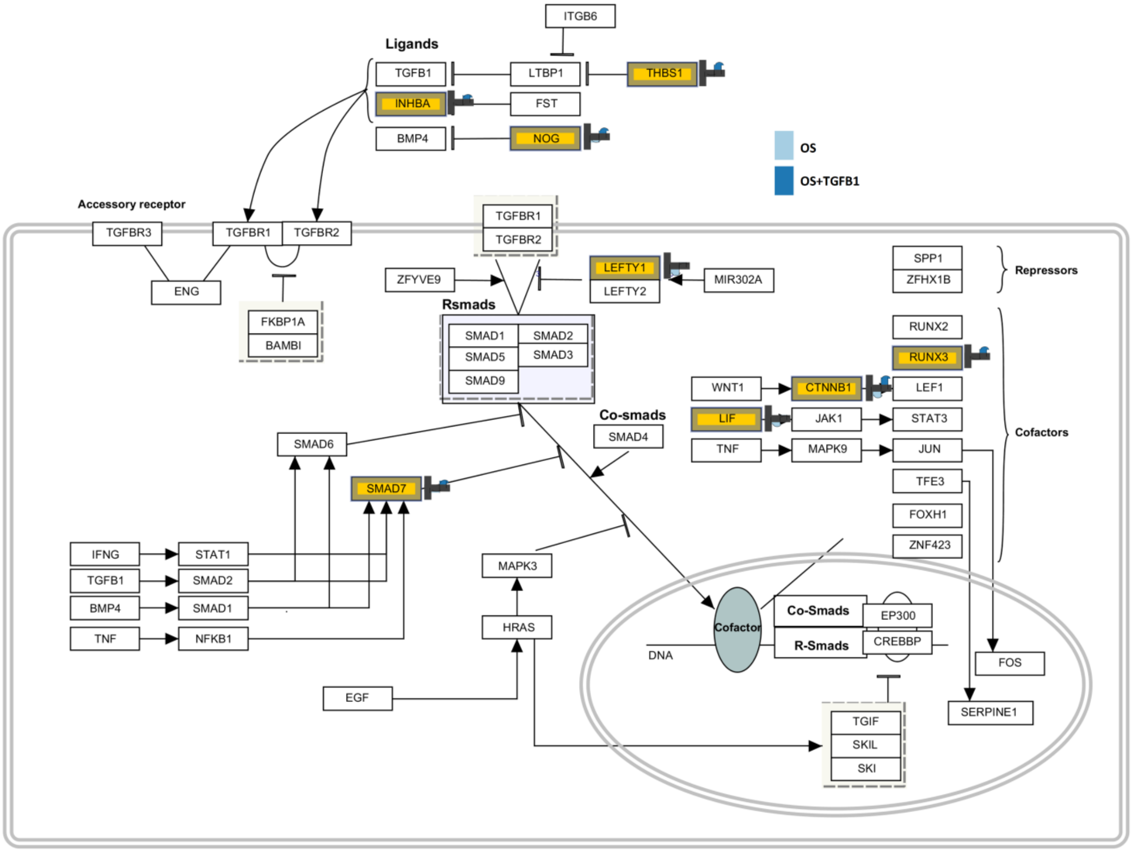

Supplementary Figure s3.

Pathway analysis of the up-regulated genes revealed significant enrichment in several pathways related to ‘TGF-β signalling’ pathways.

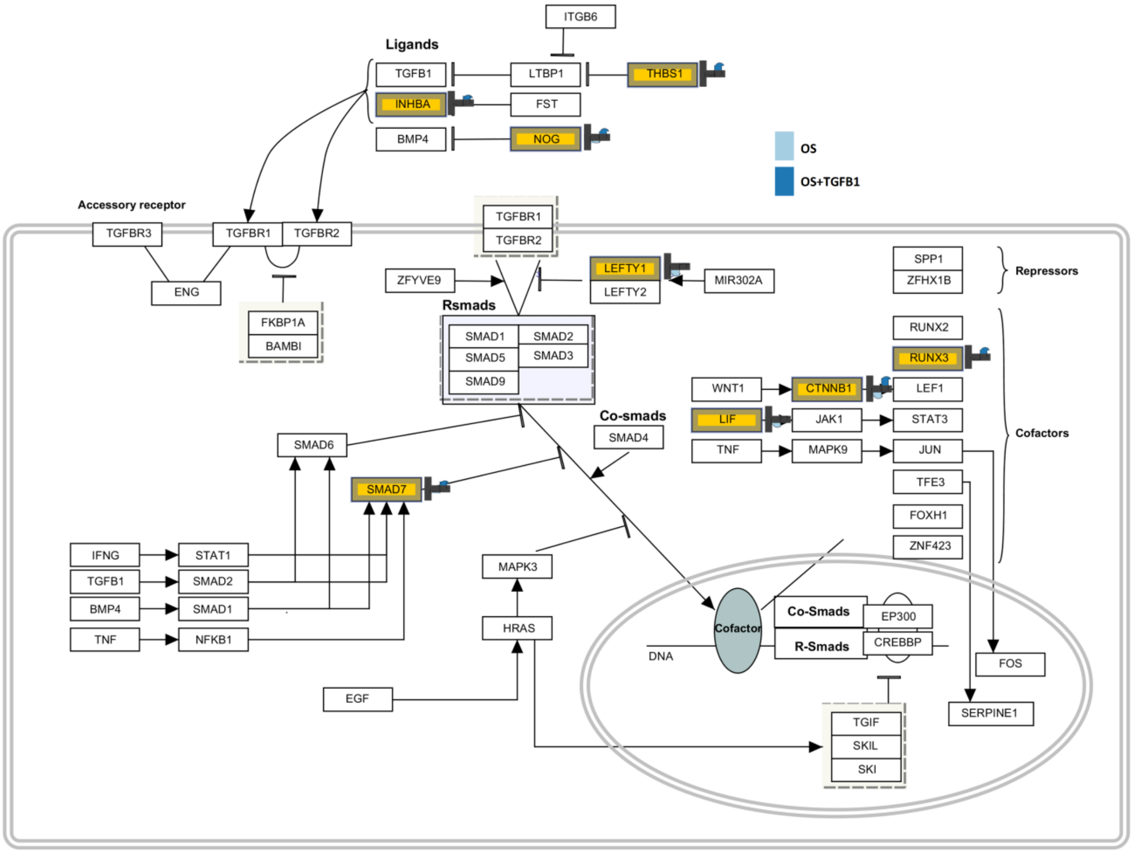

Supplementary Figure s4.

Pathway analysis of the up-regulated genes revealed significant enrichment in several pathways related to ‘WNT signalling’ pathways.

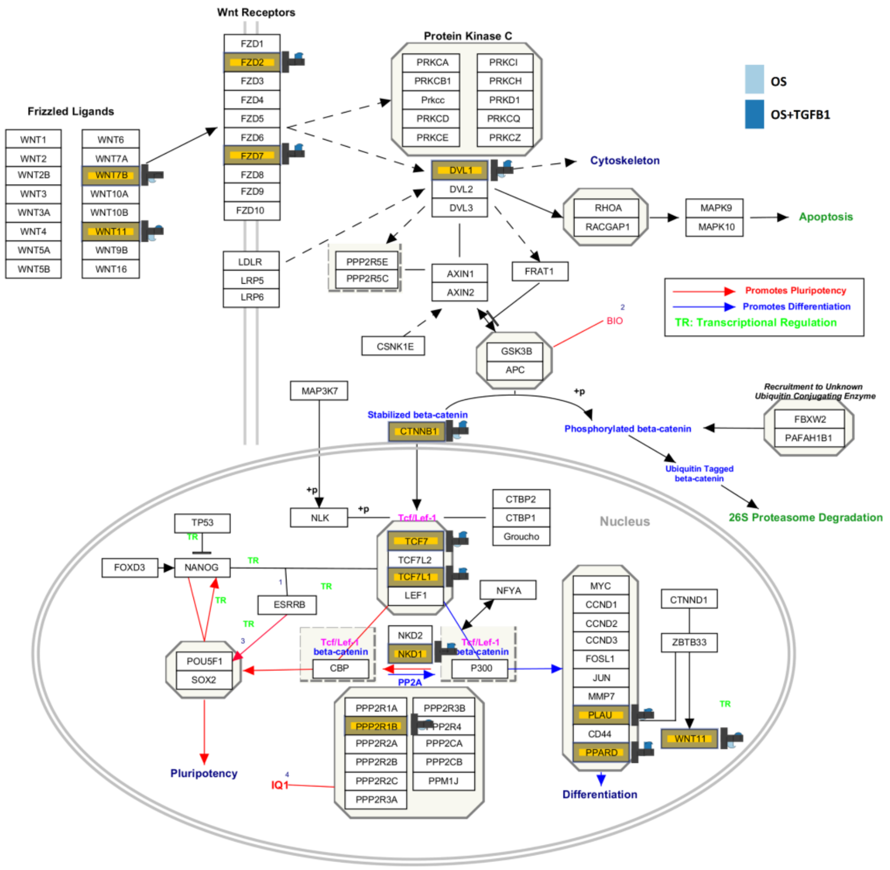

Supplementary Figure s5.

Pathway analysis of the up-regulated genes revealed significant enrichment in several pathways related to ‘MAPK signalling’ pathways.

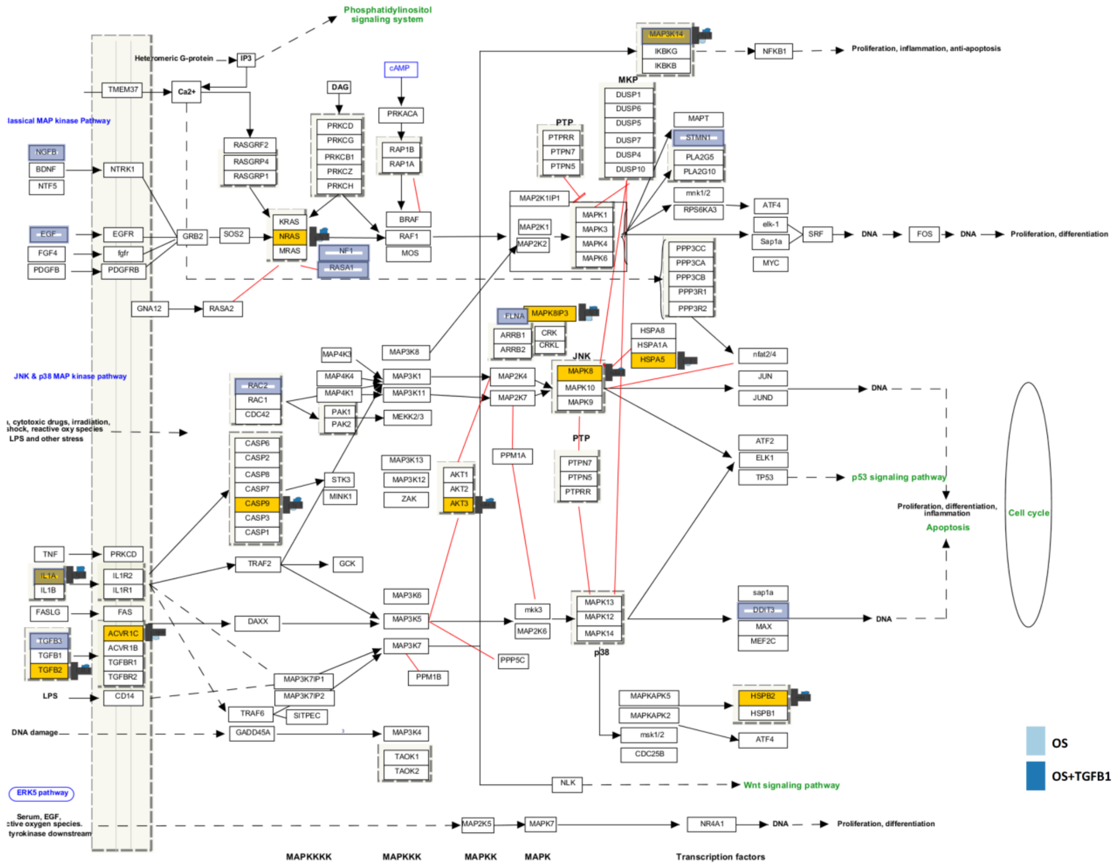

Supplementary Figure s6.

Pathway analysis of the up-regulated genes revealed significant enrichment in several pathways related to ‘focal adhesion’ pathways.

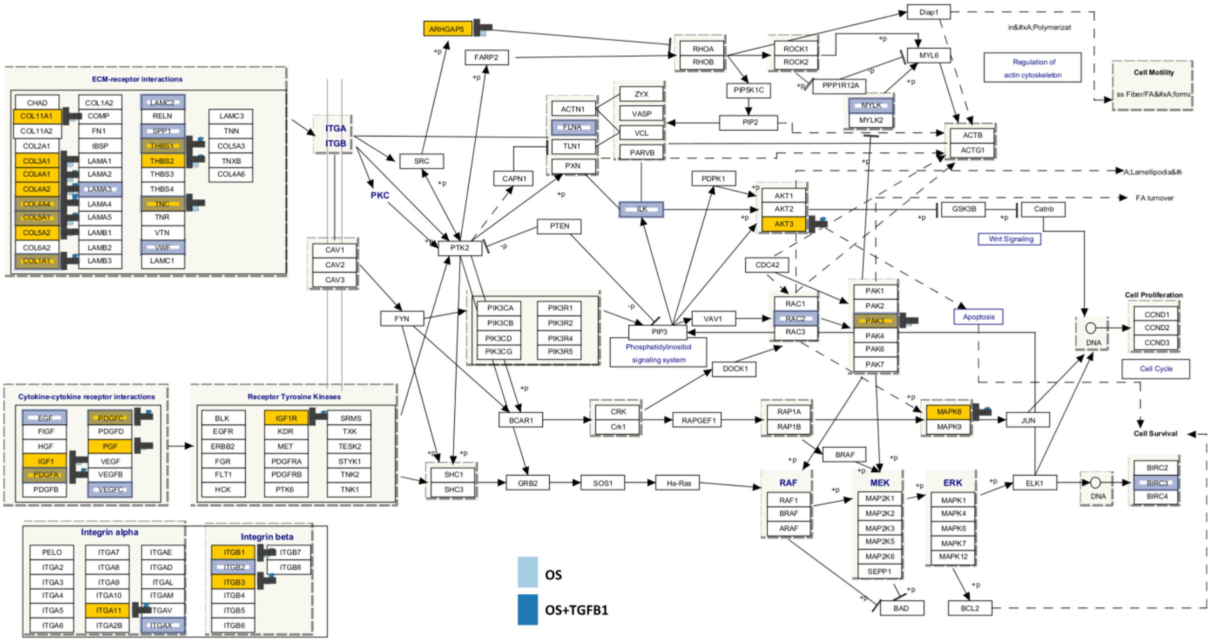

Supplementary Figure s7.

Pathway analysis of the up-regulated genes revealed significant enrichment in several pathways related to regulation of actin cytoskeleton pathways.

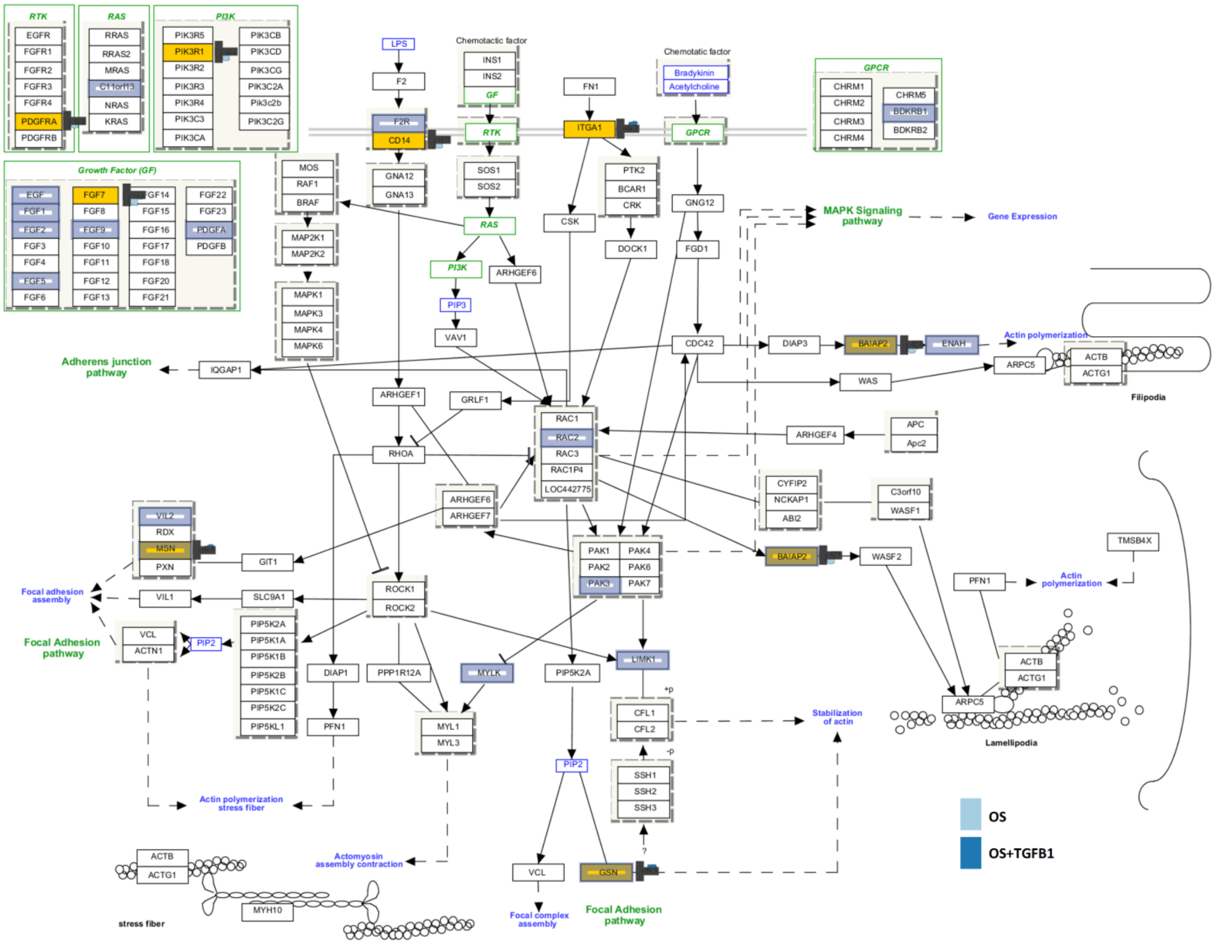

Supplementary Figure s8. TGF-β1 negatively regulates SERPINB2 in a dose and time-dependent manner during osteo- and adipocyte differentiation of hBMSC. qRT-PCR performed for SERPINB2 gene, for TGF-β1 and SB treated cells. Expression of the target gene was normalised to GAPDH. Data are shown as the SD of three independent experiments. qRT-PCR is showing time course of SERPINB2 expression between day (D)0, D1, D3, and D5 for cells induced with either osteoblast (OS) or adipocyte (AD) induction medium.

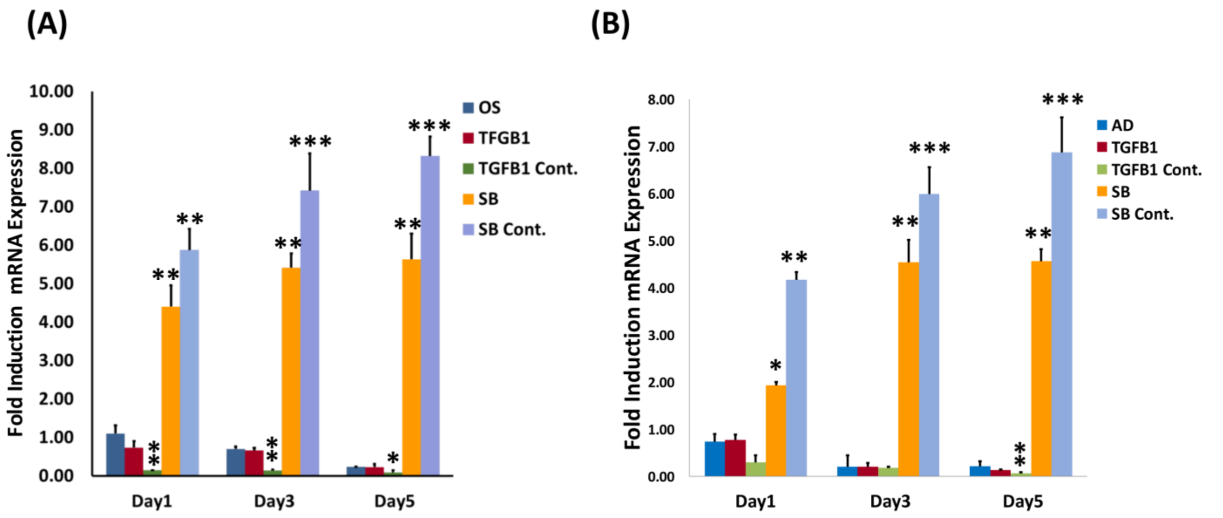

Supplementary Figure s9. Silencing of SERPINB2 in primary human bone marrow stromal (skeletal) stem cells (hBMSC). Promotes OS and AD differentiation. Primary hBMSCs were transfected with SERPINB2 siRNA or scramble siRNA (SCR) and were subjected to osteoblast or adipogenic differentiation induction (A) siRNA-mediated deficiency of SERPINB2 was confirmed by qRT-PCR which showed significant decrease in SERPINB2 in primary hBMSC. (B) Mineralized calcium deposition was determined using Alizarin Red S staining for different treatment groups. Quantification of mineralized nodules using OsteoImage Mineralization Assay. (C) Oil Red O staining of accumulated oil droplets in adipocyte induced primary hBMSC. Nile Red staining quantification. Data are presented as mean ± SD, n=3. Data are representative of at least two independent experiments, *p<0.05; ** p<0.01, ***P<0.005.

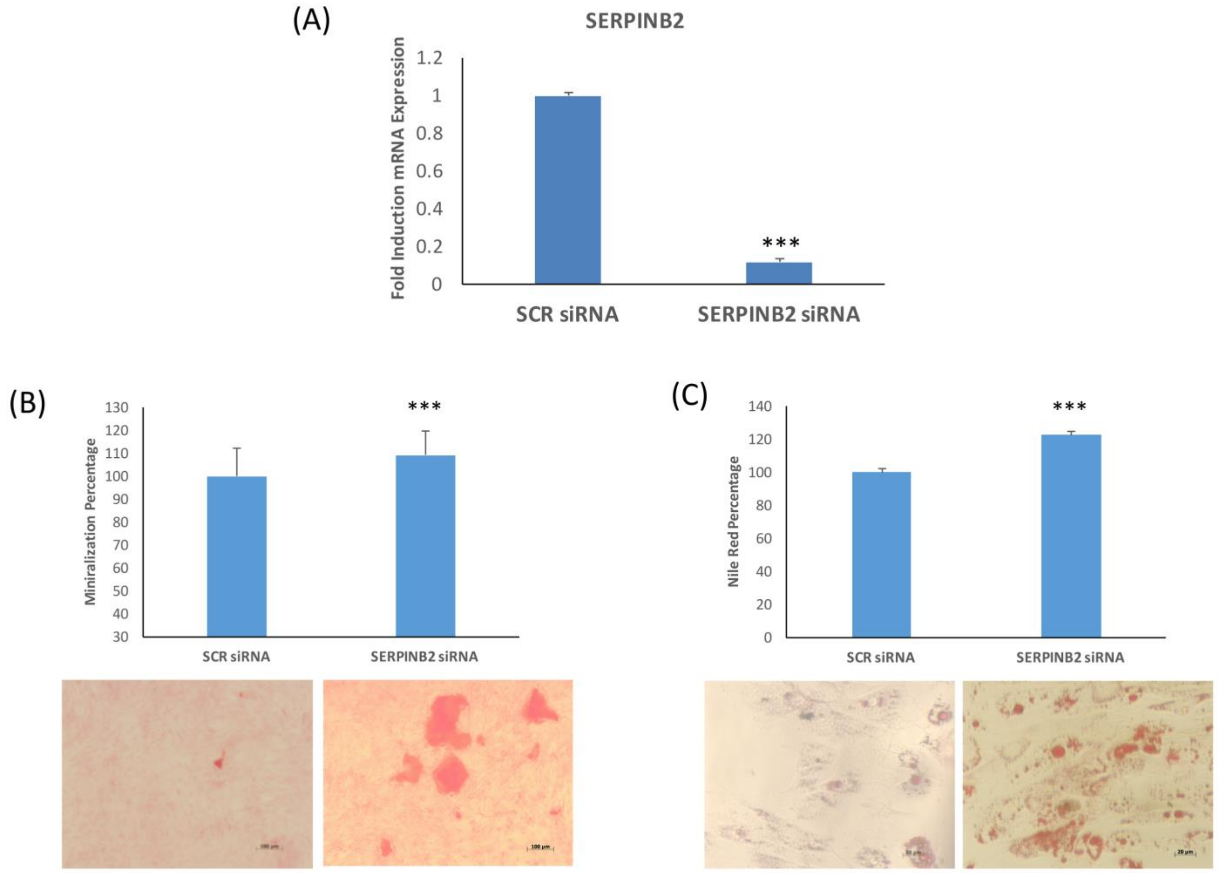

Supplementary Table S 8: Real-time PCR human primer sequences used in this study

Gene name	Forward primer (5´–3´)	Reverse primer (5´–3´)
GAPDH	CTGGTAAAGTGGATATTGTTGCCAT	TGGAATCATATTGGAACATGTAAACC
Runx2	CACCATGTCAGCAAAACTTCTT	ACCTTTGCTGGACTCTGCAC
ALPL	GACGGACCCTCGCCAGTGCT	AATCGACGTGGGTGGGAGGGG
OCN	GGCAGCGAGGTAGTGAAGAG	CTCACACACCTCCCTCCTG
PPARG	TTCTCCTATTGACCCAGAAAGC	CTCCACTTTGATTGCACTTTGG
ADIPOQ	GCAGTCTGTGGTTCTGATTCCATAC	GCCCTTGAGTCGTGGTTTCC
LPL	CTTGGAGATGTGGACCAGC	GTGCCATACAGAGAAATCTC

Supplementary Table S 9: TAQMAN Real-time PCR primers

Gene ID	Assay ID	Cat no.
GAPDH	Hs02758991_g1	4331182
SERPINB2	s10016	4392420
